# Mice with a conditional deletion of *Talpid3* (*KIAA0586*) – a model for Joubert syndrome

**DOI:** 10.1002/path.5271

**Published:** 2019-05-16

**Authors:** Andrew L Bashford, Vasanta Subramanian

**Affiliations:** ^1^ Department of Biology and Biochemistry University of Bath Bath UK

**Keywords:** Joubert syndrome, KIAA0586, primary cilia, cerebellum, sonic hedgehog, ataxia

## Abstract

Joubert syndrome (JS) is a ciliopathy associated with mutations in numerous genes encoding cilia components. TALPID3 encoded by *KIAA0856* in man (*2700049A03Rik* in mouse) is a centrosomal protein essential for the assembly of primary cilia. Mutations in *KIAA0856* have been recently identified in JS patients. Herein, we describe a novel mouse JS model with a conditional deletion of the conserved exons 11–12 of *Talpid3* in the central nervous system which recapitulates the complete cerebellar phenotype seen in JS. *Talpid3* mutant mice exhibit key hallmarks of JS including progressive ataxia, severely hypoplastic cerebellar hemispheres and vermis, together with abnormal decussation of the superior cerebellar peduncles. The Purkinje cell layer is disorganised with abnormal dendritic arborisation. The external granule layer (EGL) is thinner, lacks primary cilia, and has a reduced level of proliferation. Furthermore, we describe novel cellular defects including ectopic clusters of mature granule neurons, and abnormal parallel fibre‐derived synapses and disorientation of cells in the EGL. The defective glial scaffold results in abnormal granule cell migration which manifests as ectopic clusters of granule neurons. In addition, we show a reduction in *Wnt7a* expression suggesting that defects may arise not only from deficiencies in the Hedgehog (Hh) pathway but also due to the additional roles of *Talpid3*. The *Talpid3* conditional knockout mouse is a novel JS model which fully recapitulates the JS cerebellar phenotype. These findings reveal a role for *Talpid3* in granule precursor cell migration in the cerebellum (either direct or indirect) which together with defective Hh signalling underlies the JS phenotype. Our findings also illustrate the utility of creating conditional mouse models to assist in unravelling the molecular and cellular mechanisms underlying JS. © 2019 The Authors. *The Journal of Pathology* published by John Wiley & Sons Ltd on behalf of Pathological Society of Great Britain and Ireland.

## Introduction

The primary cilium is an antenna‐like non‐motile structure which protrudes from the cell and consists of a basal body (mother centriole) and an axoneme 1, 2. The components of the sonic hedgehog (Shh) pathway traffic through the primary cilia and transduce signals important for growth, differentiation, and morphogenesis 1, 2. In ciliopathies, cilia are either defective or absent, resulting in multiple organ defects 3, 4, 5. One such ciliopathy is Joubert syndrome (JS) [OMIM (Online Mendelian Inheritance in Man) 21330]. The central diagnostic feature of JS is the ‘molar tooth sign’ seen on MRI of the brain which results from a malorientation of the superior cerebellar peduncles (SCPs). Other brain malformations include hypoplasia of the cerebellar vermis and absence of decussation (both SCPs and the corticospinal tracts at the medullary pyramids) 6, 7, 8, 9, 10, 11. The brain malformations result in the clinical features of ataxia, abnormal eye movements, episodic neonatal breathing abnormalities, and developmental delay. In addition, JS patients can have a wide range of systemic abnormalities including retinitis pigmentosa, renal abnormalities, and polydactyly.

Mutations in around 34 genes have so far been identified as causative for JS (OMIM 21330) and a few of these are components of the basal body. One gene recently identified with mutations in Joubert and Jeune syndrome patients is *KIAA0586*, also called *TALPID3* 12, 13, 14, 15. Talpid3 is a component of the basal body and is present at the distal tip of centrosomes and associates with CP110, Cep97, Kif24, and Cep290 16. *TALPID3* is conserved in vertebrates 17 and is essential for cilia formation in chicken 18, mice 19, and zebrafish 20.

CEP290 which interacts with TALPID3 is also associated with a variety of ciliopathies with overlapping clinical features including Joubert syndrome, Joubert syndrome‐related disorders (JSRDs) 21, Meckel–Gruber syndrome (MKS) 22, and Bardet–Biedl syndrome (BBS) 23. The molecular defect in human JS/MKS caused by *CEP290* mutations includes proliferation defects of the granule cell precursors (GCPs) associated with abrogated Shh signalling 24.

Constitutive deletion of the highly conserved exons 11 and 12 of *Talpid3* in mice results in embryos with a dorsalised neural tube and embryonic lethality at E10.5, while a conditional limb‐specific deletion causes polydactyly. Both the dorsalised neural tube and the limb defects are due to abrogation of Shh signalling 19. Here, we describe the phenotype of mice with a conditional deletion of the conserved exons 11 and 12 of *Talpid3* in the brain which resembles the human JS phenotype.

## Materials and methods

### Study approval

All animal procedures were reviewed and approved by the Animal Welfare and Ethical Review Body of the University of Bath and conducted under approved Project and Personal licences in accordance with UK Home Office guidelines and the UK Animals (Scientific Procedures) Act, 1986.

### Mouse strains and breeding

*Talpid3* ^*fl/fl*^ mice 19 were maintained on a 12 h light/dark cycle with access to food and water *ad libitum*. CNS specific recombination was achieved by crossing the *Talpid3* ^*fl/fl*^ mice with the *NestinCre* line [B6.Cg‐Tg(Nes‐Cre)1kln/J] 25. Offspring were genotyped by PCR for the *Cre* transgene and the floxed and deleted *Talpid3* alleles 19.

### Histology

Postnatal mice were euthanised by injection of sodium pentobarbitone (200 mg/kg; Euthatal, Boehringer Ingelheim Animal Health, Bracknell, UK). For cell proliferation studies, mice were injected with bromodeoxyuridine (BrdU; 100 mg/kg body weight; Sigma‐Aldrich Co Ltd, Gillingham, UK) 1 h before euthanasia. Dissected brains were fixed overnight at 4 °C in 4% buffered paraformaldehyde (Sigma‐Aldrich Co Ltd), embedded in paraffin wax (Fibrowax™, VWR, Leuven, Belgium) or frozen in OCT (Tissue‐Tek®, Sakura, Japan), sectioned, and immunostained.

### Immunohistochemistry

Frozen sections were rinsed in PBS to remove OCT. Paraffin sections were dewaxed in Histoclear (National Diagnostics, Yorkshire, UK) and rehydrated. Antigen retrieval was carried in citrate buffer (10 mm sodium citrate, pH 6; 0.1% Tween‐20). Sections were blocked in blocking buffer (PBS with 0.1% gelatin, 0.5% BSA, and 0.1% Tween‐20) for 1–2 h. Sections were incubated overnight at 4 °C with the following primary antibodies: mouse anti‐calbindin (1:4000, 300; Swant, Marly, Switzerland); mouse anti‐neurofilament (1:5, 2H3; DSHB, Iowa City, IA, USA); rabbit anti‐pericentrin (1:2000, 448; Abcam, Cambridge, UK); rabbit anti‐adenylyl cyclase III (1:1000, C‐20; Santa Cruz, Dallas, TX, USA); rabbit anti‐active caspase 3 (1:2000, ab13847; Abcam); rabbit anti‐Pax6 (1:500, PRB‐278P; Covance, Dedham, MA, USA); rabbit anti‐phosphohistone 3 (1:1600, 3377; Cell Signaling Technology, Danvers, MA, USA); guinea pig anti‐VGlut (1:6000, Ab5905; Millipore, Billerica, MA, USA); rabbit anti‐GAD65/67 (1:500, AB1511; Millipore, Watford, UK); and mouse anti‐PCNA (1:4000, 2586; Cell Signaling Technology).

Sections were treated with pepsin (0.1% pepsin in 0.1 m HCl; Sigma‐Aldrich Co Ltd) for the detection of BrdU, blocked in blocking buffer, and incubated with the following primary antibody: mouse anti‐BrdU (1:50, G3G4; DSHB) overnight at 4 °C.

Unbound primary antibody was washed off in PBST (3 × 10 min) and the appropriate secondary antibody was applied to the sections and incubated for 2 h at room temperature. Secondary antibodies used for indirect immunofluorescence were rabbit anti‐mouse Alexa 568 (A11061), goat anti‐guinea pig Alexa 488 (A‐11073), and goat anti‐rabbit Alexa 488 (A‐11034), all at 1:1000 dilution (Invitrogen, Waltham, MA, USA).

Images were acquired using a Leica DM55 microscope [Leica Microsystems (UK) Ltd, Milton Keynes, UK], a DFC‐6000 camera [Leica Microsystems (UK) Ltd], and LAS software [Leica Microsystems (UK) Ltd]. Image analysis was performed using Fiji software 26.

### RNA isolation, cDNA synthesis, and RT‐qPCR analysis of signalling pathway components

Cerebella were snap‐frozen in Trizol reagent (Invitrogen) and total RNA was extracted. cDNA synthesis was performed using RevertAid H Minus Reverse Transcriptase (Thermo Fisher Scientific, Loughborough, UK). Quantitative PCR was carried out in an iQ5 thermocycler (Bio‐Rad Laboratories Inc, Watford, UK) using SYBR Green Supermix (Bio‐Rad Laboratories Inc). Expression levels were quantified relative to *Gapdh* and comparisons were made between control and mutant mice (*n* = 3 or 4). Student's *t*‐test was used to compare mean expression values between control and mutant mice. Primer sequences and product size are listed in supplementary material, Table S1.

### Western blotting

Cerebella were minced and homogenised in lysis buffer (2% SDS in 50 mm Tris, pH 6.8) and cleared lysates were snap‐frozen. Protein concentration was estimated using a BCA Protein Assay Kit (Pierce, Thermo Fisher Scientific) and proteins were resolved on a 7% Tris–glycine SDS gel (National Diagnostics) and then transferred to a PVDF membrane (Millipore). Blots were incubated overnight at 4 °C with the appropriate primary and secondary antibodies and binding was detected using the ECL chemiluminescence reaction (Pierce, Thermo Fisher Scientific) and a Vilber Lourmat Fusion imager (Collégien, France).

Details of all methods including image analysis, quantification, and statistical analysis are provided in supplementary material, Supplementary materials and methods.

## Results

### Deletion strategy and gross phenotype of *Talpid3* mutant mice

**Figure 1:**
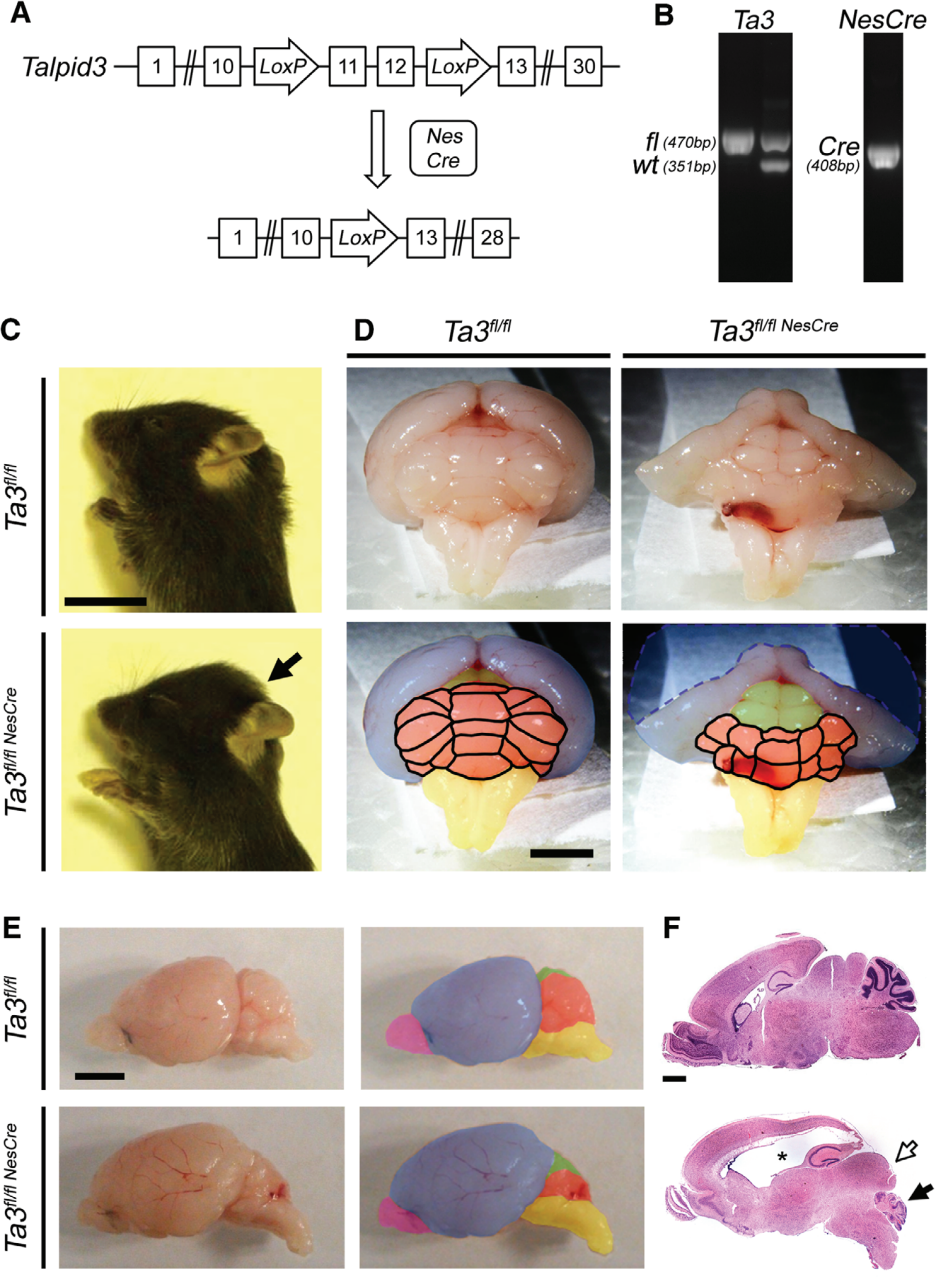
*Talpid3* knockout strategy and gross phenotype of the brain. Strategy for deletion of the coiled coil domain encoded by exons 11 and 12. The *Talpid3* ^*fl/fl*^ mice in which exons 11 and 12 are flanked by *LoxP* sequences were crossed to the *NesCre* deleter mouse, leading to the deletion of these exons. (B) PCR amplicons used to identify mice with wild‐type and *Talpid3* ^*fl/fl*^ alleles and the *NesCre* transgene. (C) P15 *Talpid3* mutant mice are smaller with a domed head (arrow). (D) Posterior view of P15 brain; *Talpid3* mutants have hydrocephaly and hypoplasia of the cerebellum. *Talpid3* mutant cerebrum appears slightly collapsed due to severe hydrocephaly indicated by the dashed blue line. (E) Lateral view of P15 whole‐mount brains and (F) sagittal sections of brain stained with H&E. Upper panel: wild type; lower panel: *Talpid3* mutant. Note the severe hydrocephaly (asterisk), posterior shift in colliculi (white arrow), and hypoplasia of the cerebellum (black arrow) in the lower panel of F. Coloured regions indicate main brain structures: cerebellum (orange), cerebrum (blue), colliculi (green), hindbrain (yellow), olfactory bulb (magenta). Scale bars: 10 mm (C); 3 mm (D, E); 2 mm (F).

We conditionally deleted the conserved exons 11 and 12 of *Talpid3* in the developing nervous system by crossing mice homozygous for the floxed *Talpid3* allele (*Talpid3* ^*fl/fl*^) to the *Nestin::Cre* (*NesCre*) deleter strain 25 (Figure 1A,B).

Since both the *Talpid3* gene and the *NesCre* transgene are located on mouse chromosome 12, we obtained a reduced frequency of mice homozygous for the *Talpid3* ^*fl/fl*^ allele carrying the *NesCre* transgene (*Talpid3* ^*fl/flNesCre*^). Throughout this paper, we will refer to the *Talpid3* ^*fl/flNesCre*^ mice as the *Talpid3* (*Ta3*) mutant mice and the *Talpid3* ^*fl/fl*^ littermates as wild type. It is noteworthy that all the *Talpid3* mutant mice that we examined consistently exhibited the phenotype that we describe here including the ataxia.

The *Talpid3* mutant mice were visually indistinguishable from their wild‐type littermates at birth, but by P15 they were smaller with a pronounced domed head (Figure 1C). The first signs of ataxia appeared at P10; ataxia progressed rapidly and was very severe by P15 (supplementary material, Video S1). However, the *Talpid3* mutant mice were still able to suckle as well as groom themselves (supplementary material, Video S1).

Macroscopic examination of the brains of P15 *Talpid3* mutant mice revealed a markedly hypoplastic cerebellum with a significantly smaller vermis and cerebellar hemispheres compared with the wild type (Figure 1D,E). These morphological abnormalities resemble the phenotype of JS/MS patients who have cerebellar hypoplasia and a markedly small or absent vermis 6, 8. The P15 *Talpid3* mutant brain also exhibited hydrocephaly, which was first detectable at P5 and progressed in severity between P5 and P15, forcing the superior and inferior colliculi into a more posterior position (Figure 1F). The hydrocephaly is unlikely to have a direct bearing on the cellular defects seen in the cerebellum as the proliferation and migration defects are already observable by P0, much before the development of hydrocephaly.

**Figure 2:**
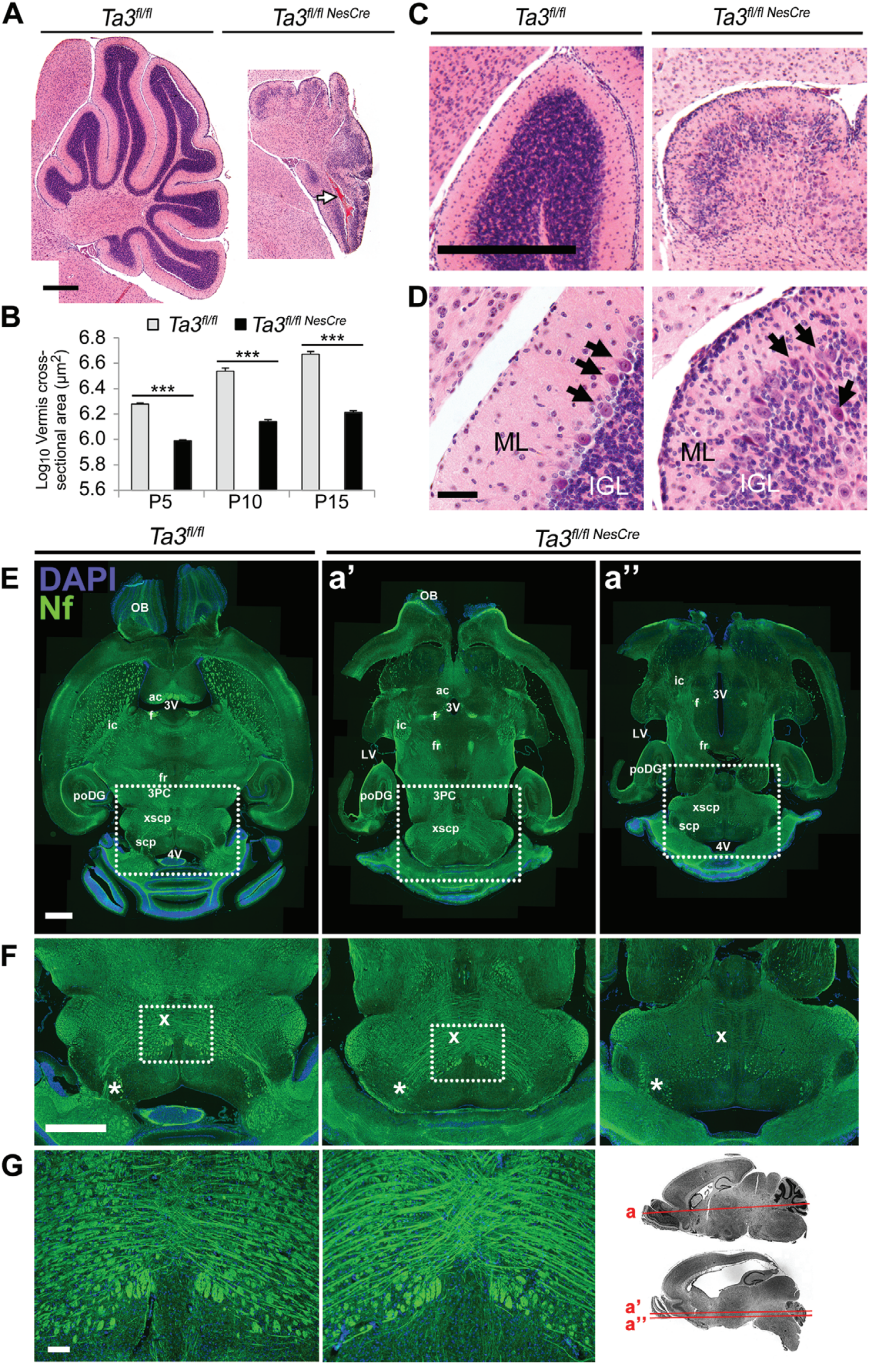
Histology of the cerebella of *Talpid3* mutant and wild‐type mice. (A) H&E‐stained cerebellar sections of wild‐type and *Talpid3* P15 mice. Note haematoma in the posterior cerebellar lobe (white arrow). (B) Cross‐sectional area of cerebellar vermis showing reduction in the *Talpid3* mutant. (C, D) Cerebellar lobe showing reduced granule neuron density in the IGL and a disorganised PCL with ectopic PCs (black arrows); some PCs appear pyknotic. *Talpid3* mutant ML also shows clusters of cells at P15 which are absent in the wild‐type ML. (E) P15 horizontal section of wild‐type and *Talpid3* mutant brain stained for neurofilament. Note the thinner and elongated midbrain of mutant mice and hydrocephaly of the lateral ventricles. (F) Greater magnification of the boxed region of the midbrain in E. Note the reduced density of tracts in the superior cerebellar peduncles, indicated by an asterisk. (G) Abnormal decussation of cerebellar peduncles with compact and thickened tracts, indicated by ‘X’. Greater magnification of the boxed region from F. Sagittal image shows the dorso‐ventral position of horizontal sections. 3PC, parvocellular oculomotor nucleus; 3V, third ventricle; 4V, fourth ventricle; ac, anterior commissure; f, fornix; fr, habenulo‐interpeduncular tract; ic, internal capsule; OB, olfactory bulb; poDG, posterior dentate gyrus; scp, superior cerebellar peduncle; xscp, decussation of the superior cerebellar peduncle. Anatomical structures were identified using The Allen Mouse Brain Library C57BL/6J horizontal atlas. Scale bars: 500 μm (A, C); 50 μm (D); 1 mm (E, F); 100 μm (G). Note: Fiji software was used to ‘stitch’ large composite pictures from overlapping images (A, E, F). Sequential images of equal exposure were taken in a grid across the region of interest with adjacent images having a small overlap. The Fiji software aligned the identical overlapping regions to stitch images together.

We corroborated the gross morphological changes seen in the brains of the *Talpid3* mutant mice with histological analysis focusing on the cerebellum. We analysed growth and fissure formation, as well as the effects on decussation of the SCPs. The five cardinal lobes of the cerebellum (anterobasal, anterodorsal, central, posterior, and inferior) and the four principal fissures arise first during the postnatal development of the cerebellum 27, 28. In contrast to the normal developmental sequence of the cardinal lobes and fissure formation seen in wild‐type mice, the *Talpid3* mutant cerebella showed a dramatic loss of foliation which was most severe in lateral regions (supplementary material, Figure S1A–E). The formation of the cardinal lobes in *Talpid3* mutant cerebella was retarded. This was first evident at E18.5, when fissures were barely visible (supplementary material, Figure S1A). There was no significant increase in size or foliation in the *Talpid3* mutant cerebella between E18.5 and P10 (supplementary material, Figure S1A–C). At P15, only the five cardinal lobes were present in the *Talpid3* mutant cerebellum and the fissures remained shallow (supplementary material, Figure S1E), suggesting a defect in proliferation and growth. The cross‐sectional area of the vermis was significantly reduced in the *Talpid3* mutant cerebella (Figure 2A,B).

The most striking feature of the *Talpid3* mutant cerebellum at P5 was the thin external granule layer (EGL), which had a lower cell density compared with the wild type (supplementary material, Figure S2A,B), and the inner granule layer (IGL) at P15 (Figure 2C, D and supplementary material, Figure S2A,D), which was sparsely populated with cells (Figure 2C,D). The integrity of the molecular layer (ML) and the IGL was lost in the P15 *Talpid3* mutant cerebella and the discrete single‐cell Purkinje cell (PC) layer was disrupted (Figure 2C,D).

We further assessed the organisation of the midbrain, the hindbrain, and the SCPs by immunostaining for neurofilament M in horizontal sections. The midbrain appeared thinner across the medio‐lateral axis (Figure 2E,F). Assessment of serial sections across the dorso‐ventral axis identified comparable regions but these were present at different axial levels due to the very large difference in shape and size of the brain. The gross organisation of the tracts in the midbrain appeared to be compressed and misshapen (Figure 2F), and the decussation of the SCPs was disrupted in the *Talpid3* mutant brain (Figure 2F,G).

### Cerebella of *Talpid3* mutant mice lack primary cilia

It has previously been shown that the deletion of the coiled‐coil domain of *Talpid3* is sufficient to disrupt cilia formation by the failure of the mother centriole to dock with the plasma membrane 18, 19. We analysed the cerebella of *Talpid3* mutant mice for primary cilia by immunostaining for adenylyl cyclase III (ACIII), a protein shown to localise at primary cilia in neurons and glial cells 29.

Primary cilia were present in the majority of cells in the EGL and IGL in wild‐type mice (supplementary material, Figure S3A,C). In contrast, cells in the cerebella of *Talpid3* mutant mice had no cilia or showed fine dots staining positive for ACIII but importantly these did not resemble primary cilia (supplementary material, Figure S3A,C). In the wild‐type EGL, 64.4% of cells possessed fully formed primary cilia, while in the mutant, 18.7% of the cells appeared to only have fine dots staining positive (supplementary material, Figure S3B). In the wild‐type IGL, fully formed primary cilia were present on 60.9% of cells compared with less than 10% of cells with dot‐like ACIII‐stained structures in the *Talpid3* mutant (supplementary material, Figure S3D).

### Effect of loss of *Talpid3* on GCPs and proliferation

GCPs originate from the rhombic lip and migrate to form the EGL of the cerebellum. The extensive proliferation of GCPs in the EGL is dependent on Shh signalling 30, 31, 32, 33, 34, 35. Post‐mitotic cells migrate tangentially and then radially inwards to the IGL. Since the *Talpid3*‐mutant EGL cells lacked primary cilia, we investigated the proliferation of GCPs in the *Talpid3* mutant mice and compared it with their wild‐type littermates in 18.5 dpc (days post coitum) embryonic and postnatal cerebellum.

GCP proliferation was assessed at E18.5, when the immature cerebellum begins its massive growth and organisation 27, 28. Cerebellar sections were stained for phosphohistone3 (PH3) and proliferating cell nuclear antigen (PCNA), and proliferating cells in each of the emerging cardinal lobes were quantified. There was a significant reduction in the number of PH3^+^ cells in four out of five regions and a reduction in PCNA^+^ cells in all regions (supplementary material, Figure S4).

**Figure 3:**
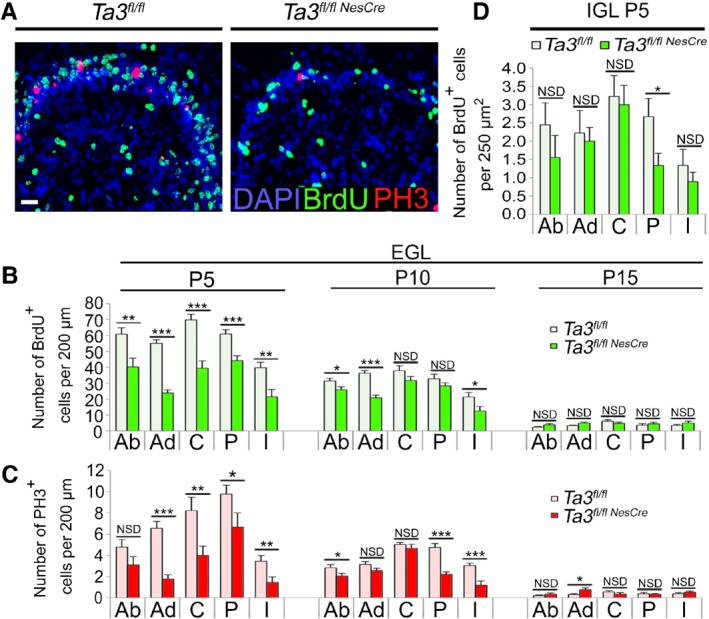
Proliferation in the EGL and IGL of *Talpid3* mutant and wild‐type cerebella. (A) Immunostained *Talpid3* P5 anterodorsal fold of the cerebellum showing BrdU^+^ and PH3^+^ cells. (B) Quantification of BrdU^+^ and (C) PH3^+^cells in the EGL of wild‐type and *Talpid3* mutant cerebella. (D) Quantification of BrdU^+^ cells in P5 EGL of wild‐type and *Talpid3* mutant cerebella. Ab, anterobasal; Ad, anterodorsal; BrdU, bromodeoxyuridine; C, central; EGL, external granule layer; I, inferior; IGL, internal granule layer; P, posterior; PH3, phosphohistone‐3. Error bars; (B, C) SEM (*n* = 3), NSD = no significant difference. ***p ≤ 0.001; **p ≤ 0.01; *p < 0.05 (one‐tailed Student's *t*‐test). (D) Box plot (*n* = 3). ***p < 0.001; **p < 0.01; *p < 0.05 (one‐tailed Mann–Whitney test). Scale bar: 50 μm (A).

We observed significantly fewer granule cells in the postnatal cerebella of *Talpid3* mutant mice and to assess if this was due to defective proliferation, we administered the thymidine analogue bromodeoxyuridine (BrdU) to *Talpid3* mutant and wild‐type sibling mice at P5, P10, and P15. Immunohistochemistry for BrdU and PH3 identified cycling cells in S‐phase or mitosis, respectively (Figure 3A). In the *Talpid3* mutant cerebella, there was a significant reduction in the number of proliferating cells in the EGL in all lobes at P5 (Figure 3B,C) and P10. By P15, proliferation was considerably reduced in both wild‐type and *Talpid3* mutant cerebella such that relative differences in proliferation observed earlier were not obvious (Figure 3B,C). The thickness of the EGL in the *Talpid3* mutant cerebellum was significantly reduced, with a lower density of cells per unit length (Figure 3C).

Concomitant with defective GCP proliferation, there was a noticeable reduction in the density of granule cells in the IGL. On average, P5 *Talpid3* mutants exhibited a 27% reduction in cell density in the IGL (Figure 3D). To assess whether the lower cell density was due to a combination of reduced cell proliferation and increased apoptosis, we immunostained cerebellar sections for cleaved caspase‐3 (supplementary material, Figure S5A,B). *Talpid3* mutants had an increased number of apoptotic cells per unit area in the EGL (supplementary material, Figure S5C) but not in the IGL (supplementary material, Figure S5D).

### Orientation and migration of GCPs

In addition to reduced proliferation and increased apoptosis, we also observed that the GCPs appeared more elliptical and were tangentially orientated in the *Talpid3* mutant EGL (supplementary material, Figure S6A). We analysed and quantified the nuclear orientation and nuclear area of cells in both *Talpid3* mutant and wild‐type EGL. In the P5 EGL of wild‐type mice, nuclei tended to orientate in a radial direction and this shifted to a tangential orientation in the granule cell‐depleted EGL by P15 (supplementary material, Figure S6B). In the P5 *Talpid3* mutant cerebellum however, EGL cells were mostly tangentially orientated and remained so until P15 (supplementary material, Figure S6B). Measurement of the nuclear area also showed that in the wild‐type EGL, the nuclei were more compact with a smaller median area (supplementary material, Figure S6C).

**Figure 4:**
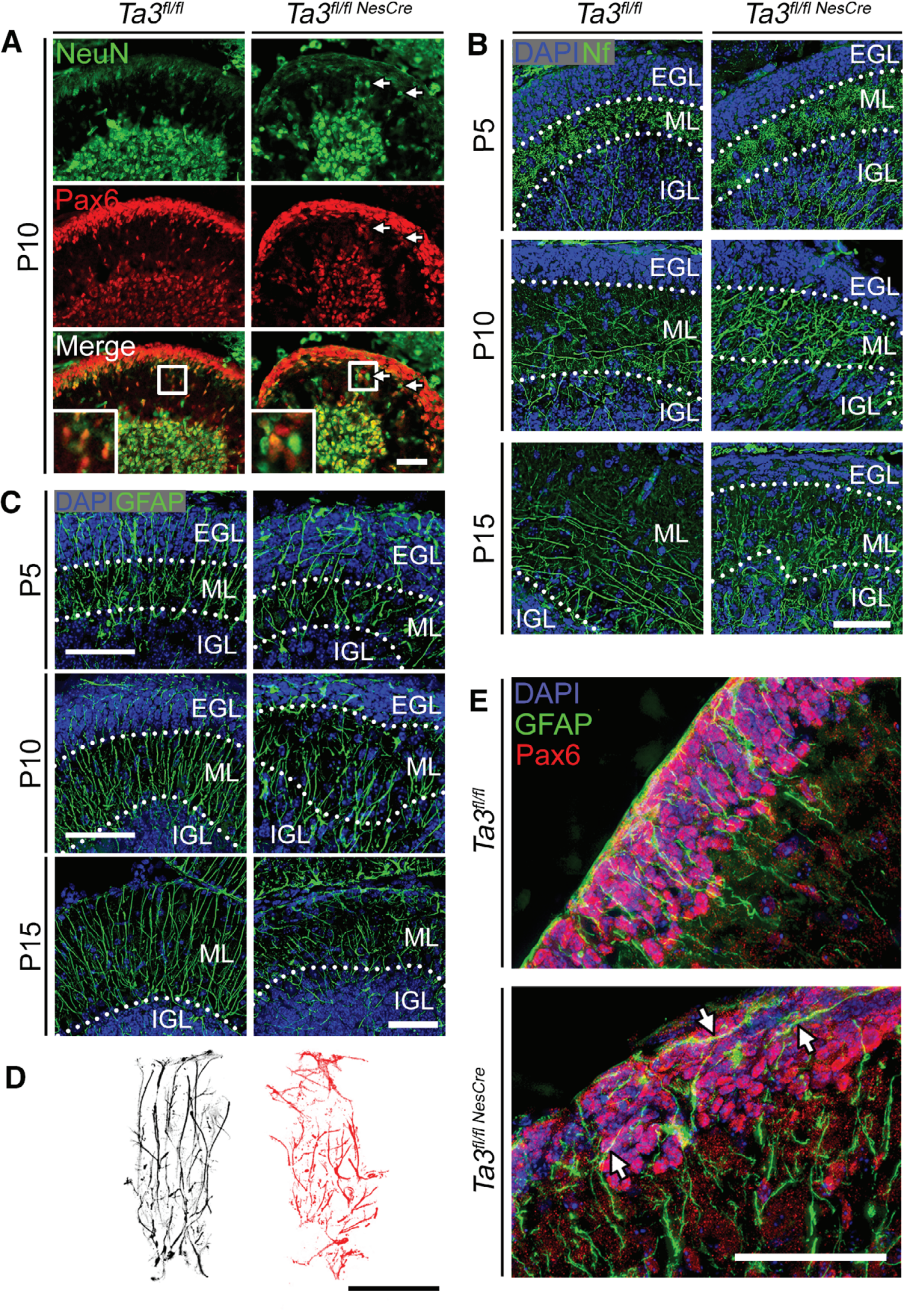
Glial scaffold and migration of GCPs in *Talpid3* mutant and wild‐type cerebella. (A) P10 wild‐type and *Talpid3* mutant cerebella immunostained for NeuN and Pax6. Arrows indicate ectopic NeuN^+^ cells in the molecular layer. The boxed region indicates enlarged area showing ectopic NeuN^+^ cells in the molecular layer. (B) P5, P10, and P15 cerebella immunostained for 165 kDa Nf. (C) P5, P10, and P15 cerebella labelled for GFAP. (D) Outline of P15 glial fibres immunostained for GFAP. (E) P10 cerebella immunostained for Pax6 and GFAP. Arrows indicate tangentially orientated glial fibres in the EGL. EGL, external granule layer; GFAP, glial fibrillary acidic protein; IGL, internal granule layer; ML, molecular layer; Nf, neurofilament. Scale bars: 50 μm.

The *Talpid3* mutant ML contained significantly large numbers of ectopic cells and its boundaries with the EGL and IGL were less defined. We identified ectopic cells by co‐immunostaining for Pax6 and the mature neuron marker NeuN. In wild‐type cerebella, GCPs in the EGL and migrating cells in the ML strongly express Pax6 and the IGL cells are negative (Figure 4A). Immature cells of the EGL and ML express very low levels of NeuN, which becomes stronger as they mature in the IGL. In the *Talpid3* mutant ML, however, we saw clusters of cells strongly expressing NeuN with barely detectable to low levels of Pax6, indicating that they were maturing/mature granule neurons (Figure 4A). It is likely that these ectopic NeuN‐positive cells have undergone premature maturation in the ML before reaching the IGL due to defective migration.

To further characterise the organisation of the ML, we stained for the 165 kDa neurofilament M, which identifies axonal tracts. At P5, the organisation of the axonal tracts appeared similar between wild‐type and *Talpid3* mutant, with long axons originating from the central nuclei extending radially through the IGL but which were denser in the *Talpid3* mutant cerebella (Figure 4B). By P10, the axonal tracts in the ML of the wild‐type cerebellum (Figure 4B) extended both tangentially and radially in the basal half of the ML. In contrast, the axonal tracts in the *Talpid3* mutant ML extended predominantly in the radial axis, which became more pronounced by P15 in the *Talpid3* mutant (Figure 4B).

### Glial scaffold defects

Bergmann glia form the scaffold on which the granule neurons migrate to the IGL. To assess the morphology of the glial scaffold, we stained sections of the cerebellum for GFAP at P5, P10 and P15. In wild‐type cerebella, the glial scaffold consisted of parallel well‐organised fibres which increased in density by P15 (Figure 4C,D). In the *Talpid3* mutant cerebella, there was a reduction in the number of Bergmann glia and a concomitant loss in the density of glial fibres extending through the ML (Figure 4C,D). The *Talpid3* mutant glial scaffold was not only less dense but also disorganised, with more tangentially orientated fibres (Figure 4C,D).

Bergmann glia were further identified by immunostaining their radial fibres for nestin and their cell soma with BLBP (supplementary material, Figure S7). In the wild‐type P5 cerebellum, the position of the cell soma was in the boundary between the IGL and ML, whereas in the *Talpid3* mutant, numerous cell bodies were mislocalised throughout the ML (supplementary material, Figure S7A) and this was more prominent by P15 (supplementary material, Figure S7B). Interestingly, the misorientation of granule cell nuclei correlated with an increased number of tangentially orientated glial fibres in the EGL. Double labelling for the granule precursor marker Pax6 and the glial protein GFAP at P10 clearly showed neat parallel glial fibres in the wild‐type EGL but the *Talpid3* mutants exhibited an aberrant organisation with more tangential fibres lying adjacent to tangentially orientated Pax6‐positive GCPs (Figure 4E).

### The Purkinje cell layer is disorganised with abnormal PCs

In wild‐type mouse cerebella, the PCs start expressing Shh when they integrate into the PCL and this acts as a potent mitogen leading to massive proliferation of the GCPs 30, 31, 32, 33, 34, 35. In H&E‐stained sections of the wild‐type cerebellum, the PCs formed a distinct monolayer surrounding the internal granule layer (IGL), which was disorganised in the *Talpid3* mutant cerebellum with heterotopic PCs (Figure 2D). To investigate this further, we stained PCs with an antibody to calbindin D‐28k 36, a calcium binding protein, which confirmed the presence of a disorganised PCL. In addition in the *Talpid3* mutant, groups of PCs were present at the base of the folds as well as in the centre of the IGL and ML (supplementary material, Figure S8A,B).

The *Talpid3* mutant cerebella, although smaller than the wild type, had a surprisingly high density of PCs. The *Talpid3* mutant P10 cerebellum had a 23% reduction in the total number of PCs but a density approximately twice that of wild type (supplementary material, Figure S8C,D). Since there was an apparent loss of PCs in the *Talpid3* mutant mice, we investigated if the reduction in PC numbers was due to increased cell death. Staining for the apoptotic marker cleaved caspase‐3 together with calbindin D‐28k did not detect any apoptotic PCs in the wild‐type or mutant cerebella at P10 (supplementary material, Figure S3A).

The dendritic arborisation of the PCs in the *Talpid3* mutant cerebella exhibited asymmetry and disorganisation. The primary dendrite that extended from the cell body to the first bifurcation branched prematurely (supplementary material, Figure S8E–G). In addition, a small number of PCs appeared to be completely misorientated, extending processes in the wrong direction (supplementary material, Figure S8E). This correlated with the loss of the discrete layering in the cerebellar cortex and the lack of postnatal growth. In the wild‐type cerebellum, the PCs displayed a characteristic pattern of dendrites with a clear primary branch which bifurcated to eventually form the numerous spiny branches at the distal tips. In the *Talpid3* mutant cerebella, the PCs had a shorter primary dendrite (supplementary material, Figure S8E–H) which branched into fine structures more similar to the spiny distal tips (supplementary material, Figure S8E,G).

### Effects of *Talpid3* loss on cerebellar circuits

The cerebellum modifies and coordinates motor signals and undergoes the majority of its growth postnatally as animals learn to move 27, 28 and PCs constitute the principal neuron in cerebellar circuits. Granule neurons, the presynaptic afferents of the PCs, have previously been shown to be potent regulators of PC development 37. Since *Talpid3* mutants are ataxic and we saw aberrations in GCP proliferation and organisation of the ML, IGL, the PCs, and their dendrites, we investigated if there were defects in cerebellar circuit formation. We visualised the different subsets of nerve terminals on the dendrites of PCs by immunostaining with antibodies directed against distinct nerve terminal‐associated proteins.

**Figure 5:**
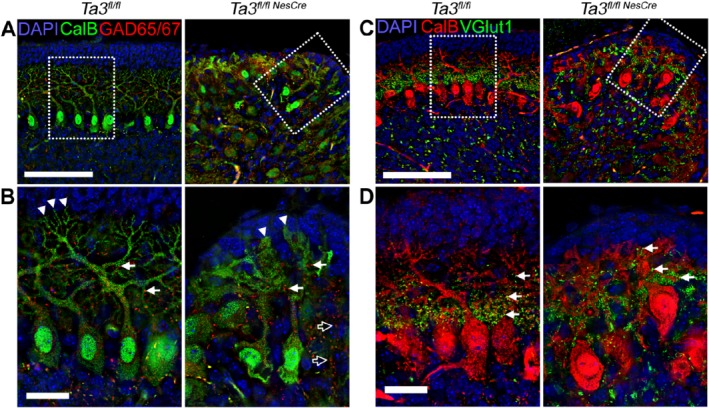
Inhibitory and excitatory synapses in Purkinje neurons on *Talpid3* mutant and wild‐type mice. (A, B) P10 wild‐type and *Talpid3* mutant cerebellar sections immunostained with anti‐GAD‐65/67 for inhibitory synapses. The box indicates region of greater magnification (B). (C, D) P10 wild‐type and *Talpid3* mutant cerebella immunostained with anti‐VGlut1 for excitatory synapses at Purkinje cell dendrites. The box indicates region of greater magnification (D). CalB, calbindin; GAD, glutamic acid decarboxylase; VGlut1, vesicular glutamate transporter 1. Scale bar: 100 μm (A, C); 25 μm (B, D). White arrow heads‐dendritic spines; white arrows ‐ synapses; Outlined black arrows ‐ abnormally placed synapses.

Glutamic acid decarboxylase (GAD‐65/67), the enzyme responsible for GABA synthesis, is a marker for presynaptic inhibitory terminals originating from stellate and basket interneurons. Inhibitory terminals were identified by labelling with antibodies against GAD‐65/67. In both wild‐type and *Talpid3* mutant PCs, GAD‐65/67‐positive synapses were present throughout the dendritic arbors (Figure 5A,B) and the overall density and distribution of inhibitory synapses were comparable.

Excitatory synapses formed by the climbing fibres and granule cell parallel fibres (PFs) were labelled with antibodies against vesicular glutamate transporter 1 (VGlut1) 38. In the cerebella of wild‐type mice, the excitatory synapses were clustered at primary dendrites of the PCs but in the *Talpid3* mutant this was disrupted and excitatory synapses were seen throughout the dendritic arbors extending into the ML as well as the EGL (Figure 5C,D). This suggests that the cerebellar circuitry is significantly disrupted in the *Talpid3* mutant mice.

### Signalling pathways in *Talpid3* mutant mice

**Figure 6:**
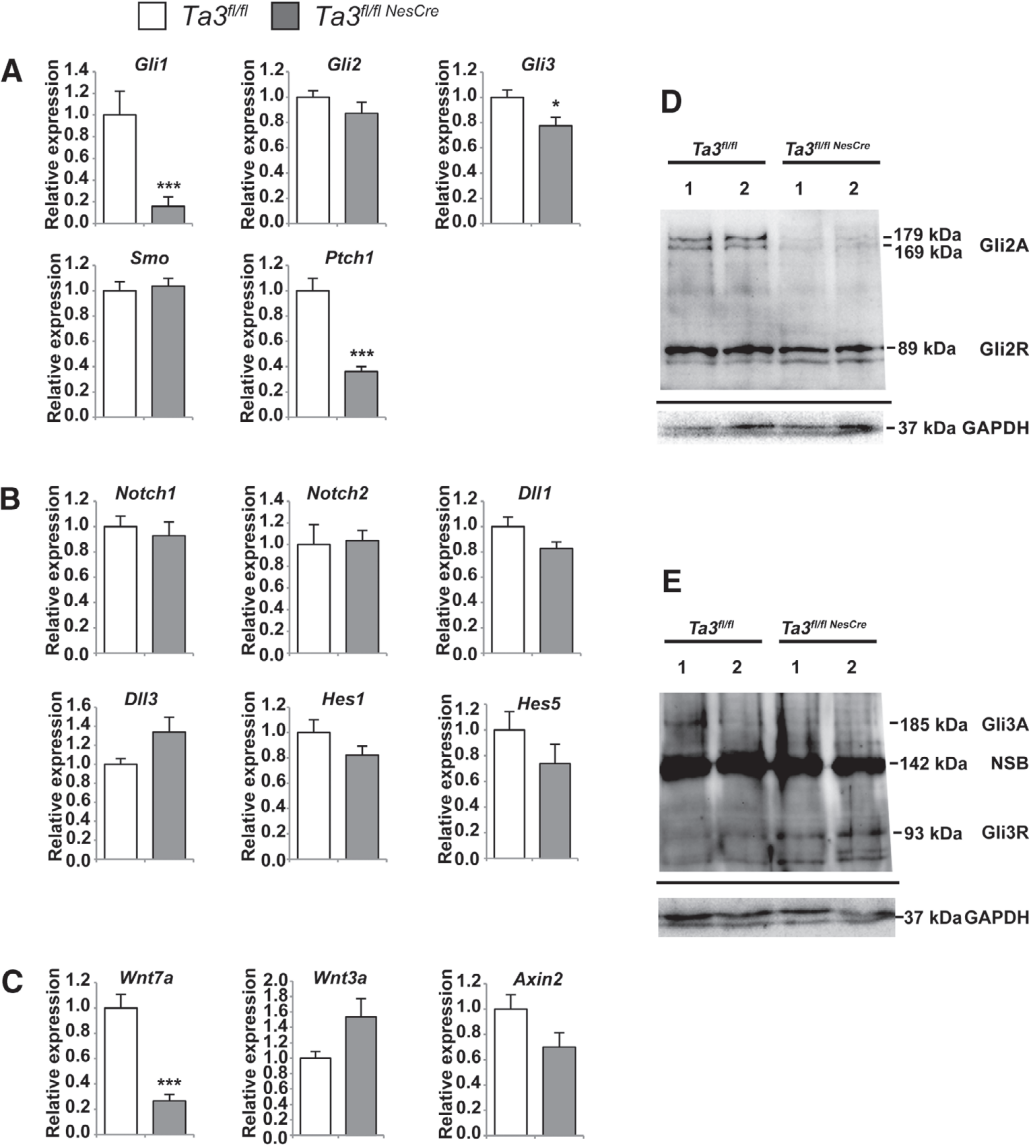
Shh, Notch, and Wnt pathways in *Talpid3* mutant and wild‐type mice. Relative mRNA expression of Shh, Notch, and Wnt pathway components in the cerebella of wild‐type and *Talpid3* mutant mice: (A) *Gli1*, *Gli2*, *Gli3*, *Smo*, and *Ptch1*; (B) *Notch1*, *Notch2*, *Dll1*, *Dll3*, *Hes1*, and *Hes5*; (C) *Wnt7a*, *Wnt3a*, and *Axin2*. Mutants show a significant reduction in the expression of *Gli1*, *Gli3*, *Ptch1*, and *Wnt7a*. Expression levels were calculated relative to *Gapdh*. Error bars (A–C) SEM (*n* = 3 or 4); ***p ≤ 0.001, *p < 0.05 (two‐tailed Student's *t*‐test). Western blot of P15 cerebellar extracts showing the processing of (D) Gli2 and (E) Gli3 in *Talpid3* mutant and wild‐type mice. The data shown are from two mice each for wild type and mutant (indicated by 1, 2). (D) *Talpid3* mutant lacks the two high‐molecular‐weight bands (179, 169 kDa) corresponding to Gli2A seen in the wild‐type siblings. (E) Gli3 appears as a faint band in one wild‐type sample (185 kDa) corresponding to Gli3A and is absent in both mutant mice. Mutant mice show a strong 93 kDa band corresponding to Gli3R. The non‐specific band seen at 142 kDa is consistent with previously published literature. The migrating dye front is seen as a faint band below the Gapdh loading control.

Primary cilia are known to be essential for hedgehog signalling 34, 35 and it has been shown by Aguilar *et al* that levels of *GLI1* and *PTCH1* mRNA are significantly reduced in most cases of JS 24. We assessed the effects of loss of *Talpid3* and cilia on Shh pathway components in the cerebella of *Talpid3* mutant mice by RT‐qPCR. The expression levels of *Gli1* and *Ptch1*, the target genes of the Hh pathway in the GCPs, were significantly reduced in *Talpid3* mutant mice (Figure 6A).

We also investigated the expression levels of the components of the Notch signalling pathway (*Notch1*, *Notch2*, *Dll1*, *Dll3*) and Notch‐responsive genes (*Hes1*, *Hes5*) by RT‐qPCR, but no significant differences were observed between *Talpid3* mutant and wild‐type siblings (Figure 6B). The Wnt signalling pathway has been suggested to play an important role in cerebellar development. Since we see aberrations in cerebellar patterning in the *Talpid3* mutant mice, we analysed the expression of *Wnt3a* and the Wnt‐responsive gene *Axin2* but found no significant changes in their expression levels compared with wild‐type sibling mice. However, we did see a considerable reduction in the expression of *Wnt7a* (Figure 6C) in the *Talpid3* mutant cerebella.

To further assess the defect in Shh signalling, processing of both Gli2 and Gli3 proteins was analysed by western blotting of cerebellar extracts from *Talpid3* mutant and wild‐type sibling mice. Analysis of Gli2 protein detected two high‐molecular‐weight bands at 179 and 169 kDa and a lower band at 89 kDa, which correspond to Gli2 activator and Gli2 repressor, respectively, in the wild‐type mice. The *Talpid3* mutant mice showed a consistent reduction in Gli2A bands (Figure 6D) when compared with the wild‐type sibling.

Analysis of Gli3 protein showed similar results; in all wild‐type sibling mice, a faint band was observed at 185 kDa and one at 93 kDa, corresponding to Gli3 activator and Gli3 repressor, respectively. A non‐specific band was also seen at 142 kDa, which is consistent with previous studies using the anti‐Gli3 antibody 39. The level of Gli3A was low in mice of both genotypes; however, in the cerebella of *Talpid3* mutant mice, there was a noticeable increase in the level of Gli3R (Figure 6E).

## Discussion

We created a CNS‐specific conditional knockout mouse for *Talpid3* since the constitutive *Talpid3* mutant mouse is embryonic lethal (E9.5–10.5) 19. We investigated if its phenotype resembled JS as mutations in *Talpid3* have been identified in JS patients. Deletion in *Talpid3* leads to a loss of primary cilia, which are essential for Shh signalling (refs 18, 19, 20 and this study). Here, we show for the first time that a conditional deletion in *Talpid3* in the CNS of mice recapitulates the human JS phenotype such as ataxia, hypoplastic cerebellar hemispheres, small vermis, and abnormal decussation of the SCPs. A limb‐specific knockout described in a previous study has polydactyly 19, another feature which is sometimes found, although not essential for the diagnosis of JS.

Mouse mutants have been generated for a few JS‐causative genes such as *Cep290*, *Ahi1*, *Cep41*, *Tmem67*, *Rpgrip1l*, and *Cep120* 40, 41, 42, 43, 44, 45, 46. Most of these mice are constitutive knockouts or gene‐trapped. The phenotypes reported for some of these mutants are at embryonic stages (when the cerebellum is either not developed or fully formed) and in other cases do not fully manifest all the features of JS 40, 41, 42, 43, 44, 45, 46. Unlike the conditional *Talpid3* mutant mice, the cerebellum was not severely affected in the *Ah1* and *Cep290* mutant mice described by Lancaster *et al* 40. The brain in the *Ah1* mutants was slightly smaller with mild foliation defects in the cerebellum, whereas in the *Cep290* mutants, only foliation was mildly affected. In addition, both mutant mice had midline fusion defects, which in the case of the *Ah1* mutant were attributed to defective Wnt signalling 40. In another mouse mutant for *Cep290* generated by gene trapping, the cerebellum appeared normal but the brain developed hydrocephaly 41.

The *Talpid3* mutant mice develop many of the histological and cellular features of human JS such as loss of integrity of the PCL, presence of heterotopic PCs, and ectopic mature neurons in the ML 24. At the molecular level, there is a significant reduction in the expression levels of *Gli1* and *Ptch1* in the *Talpid3* mutant cerebella and defective processing of Gli2 and Gli3 with aberrant Shh signalling similar to that seen in human JS/MKS cerebella 24. The severe cerebellar phenotype seen in the *Talpid3* mutant can be attributed to an increase in Gli2 and Gli3 repressors. This is in contrast to the *Cep290* and *Ahi1* mutant mice 9, 40 in which defects in GCP proliferation or effects on Shh pathway components were not detected in later stages of hindbrain development.

A conditional mouse mutant for the Talpid3 interacting protein Cep120 has been reported which lacks primary cilia and has a hypoplastic cerebellum with reduced GCP proliferation 46. However, the Purkinje cell layer does form normally, unlike in the *Talpid3* mutant. In addition, loss of *Cep120* caused centriole duplication, which we did not observe in the *Talpid3* mutant. Mouse mutants of the intraflagellar transport proteins Kif3a and Ift88 exhibit defective GCP proliferation, a disrupted PCL, and aberrant development of Bergmann glia resulting from the inability to respond to Shh signalling 34, 35, similar to that seen in the *Talpid3* mutant. The loss of *Kif3a* results in a significant reduction in *Gli1* expression, also seen in the *Talpid3* mutant 34.

Besides Shh, other signalling pathways have also been suggested to act through primary cilia and include the Wnt and Notch pathways 10, 47. Wnt signalling is important in early cerebellar development; however, in the *Talpid3* mutant cerebella there was no significant effect on the expression levels of *Axin2*, a well‐known negative regulator of Wnt signalling which is upregulated upon activation of the canonical Wnt pathway 48. Interestingly, we did observe a reduction in *Wnt7a* expression in the *Talpid3* mutant cerebella. *Wnt7a* is implicated in granule cell development. It is expressed postnatally in the IGL and in cell cultures has been shown to play a role in axonal migration and branching 49. It is tempting to speculate that the reduction in *Wnt7a* expression levels seen in the mutant cerebellum may underlie the maturation and migration defects seen in the granule neurons. Another possible explanation for the reduction in *Wnt7a* expression is that the loss of granule neurons results in a smaller number of cells expressing *Wnt7a*, thus affecting the expression levels. At present, it is unclear whether the effect on *Wnt7*a expression is a cause or a consequence of the deletion of *Talpid3* exons 11–12.

In addition to the reduction in GCP proliferation caused by the loss of primary cilia and Shh signalling, a consistent defect seen in the *Talpid3* mutant cerebella was the aberrant migration of GCPs. This manifested as misorientated GCPs, many of which failed to reach the IGL. These migration defects coincide with an aberrant scaffold formed by the Bergmann glia. Migration defects of *talpid* ^*3*^ mutant cells were first described in an explant culture of chick limb mesenchymal cells 50. The saltatory migration of *talpid* ^*3*^ mutant cells had greater periods of rest rather than a defective rate of migration. Yin *et al* found that *talpid* ^*3*^ mutant cells in culture exhibited slower microtubule regrowth 18, which may explain the migration defects. Presumably, this could be due to the role of the centriole as a microtubule‐organising centre in the cell. A scenario is possible in which slow‐moving cells simply become trapped in the rapidly developing matrix, and this might explain the migration defects that result in the ectopic cells seen in the *Talpid3* mutant ML. This defect may be exacerbated by the defective glial scaffold. Stephen *et al* 12 have also shown polarity defects in the neuroepithelium of the *talpid* ^*3*^ chick, suggesting that Talpid3 has other functions in addition to its role in cilia formation.

The *Talpid3* constitutive knockout mouse is embryonic lethal (mice survive until E10.5) 19 but mice with conditional deletion of exons 11 and 12 in the CNS survive to postnatal stages because the deletion is CNS‐specific. It is unclear if the deletion of exons 11 and 12 of *Talpid3* leads to the production of a protein lacking the region encoded by these exons. This could be verified only if we had access to an anti‐Talpid3 antibody with greater specificity. Some of the mutations in *TALPID3* found in the compound heterozygote JS patients are in exons that are transcript‐specific and others are splice site mutations; therefore, it has been suggested that the mutant TALPID3 retains some of its function in JS patients, which may explain why there is no embryonic lethality 14. Mutations in *TALPID3* have also been found in lethal ciliopathies that are inherited in an autosomal recessive manner. In those cases, one of the mutations reported caused a nonsense‐mediated decay of transcripts with exon 2, and the other the production of a transcript without exon 14 and an altered reading frame 51. The severity of the phenotypes caused by mutations in *TALPID3* thus appears to depend on the type of mutation and the transcript that is affected.

In summary, the hypoplastic cerebellum caused by reduced proliferation and survival of GCPs is one of the most striking developmental phenotypes of the *Talpid3* mutant mice. This can be attributed to the loss of primary cilia and compromised Shh signalling. *Talpid3* has additional roles in cell migration and orientation. Deletion in *Talpid3* affects cell migration, which disrupts the cellular organisation of the cerebellum. This in turn affects the cerebellar circuitry, which is the likely cause of ataxia. The *Talpid3* conditional deletion mutant mouse recapitulates the hallmarks of Joubert syndrome. This, taken together with the identification of mutations in *KIAA0586* (*TALPID3*) in some JS patients, establishes the *Talpid3* CNS conditional mutant mouse as a model for JS.

## Author contributions statement

ALB and VS designed the experiments and VS supervised the project. AB performed the experiments. ALB and VS analysed the data and wrote the manuscript.

**SUPPLEMENTARY MATERIAL ONLINE**

**Supplementary materials and methods**

**Supplementary video legend**

**Figure S1**. Growth and foliation of cerebella in *Ta3* mutant and wild‐type mice

**Figure S2**. Cell density and features of the EGL and IGL of the *Ta3* mutant and wild‐type cerebella

**Figure S3**. Primary cilia in *Ta3* mutant and wild‐type cerebella

**Figure S4**. Cell proliferation in E18.5 *Ta3* mutant and wild‐type cerebella

**Figure S5**. Apoptosis in the cerebella of *Ta3* mutant and wild‐type mice

**Figure S6**. Orientation of nuclei/cells in *Ta3* mutant EGL

**Figure S7**. Bergmann glia are misplaced in the *Ta3* mutant cerebellum

**Figure S8**. Morphology of the PCL, PCs, and dendritic arborisation in *Talpid3* mutant and wild‐type cerebella

**Table S1**. Primer sequences for qPCR

**Video S1**. *Ta3* mutant mice exhibit severe ataxia but maintain grooming behaviour

## Supporting information


**Supplementary materials and methods**

**Supplementary video legend**

**Figure S1**. Growth and foliation of cerebella in *Ta3* mutant and wild‐type mice
**Figure S2**. Cell density and features of the EGL and IGL of the *Ta3* mutant and wild‐type cerebella
**Figure S3**. Primary cilia in *Ta3* mutant and wild‐type cerebella
**Figure S4**. Cell proliferation in E18.5 *Ta3* mutant and wild‐type cerebella
**Figure S5**. Apoptosis in the cerebella of *Ta3* mutant and wild‐type mice
**Figure S6**. Orientation of nuclei/cells in *Ta3* mutant EGL
**Figure S7**. Bergmann glia are misplaced in the *Ta3* mutant cerebellum
**Figure S8**. Morphology of the PCL, PCs, and dendritic arborisation in *Talpid3* mutant and wild‐type cerebella

**Table S1**. Primer sequences for qPCR

**Video S1**. *Ta3* mutant mice exhibit severe ataxia but maintain grooming behaviour
