# Supplementary material for: Mice with a conditional deletion of Talpid3 (KIAA0586) – a model for Joubert syndrome
Source: J Pathol. 2019 May 16;248(4):396–408. doi: 10.1002/path.5271 (PMC6767539; doi:10.1002/path.5271)
Supplement: Supplementary file 1 — Supplementary materials and methods [file PATH-248-396-s001.docx]

**Mice with a conditional deletion of *Talpid3* (*KIAA0586*) – a model for Joubert syndrome**

Bashford AL *J Pathol* DOI: 10.1002/path.5271

**Supplementary materials and methods**

Reference numbers refer to the main text reference list

**Histology**

BrdU-incorporated cerebella were dissected and fixed overnight in ice-cold methanol–acetic acid (3:1) overnight. Methanol–acetic acid-fixed brains were dehydrated through ethanol series, cleared in toluene, and embedded in Fibrowax™ (VWR International, Leuven, Belgium). Sections of 10 µm thickness were cut using a microtome (Leica Jung RM2035; Leica Microsystems Ltd, Milton Keynes, UK).

PFA-fixed brains were washed in PBS, placed in cryoprotectant (30% sucrose, 0.05% sodium azide in PBS) at 4°C until fully infiltrated (1–2 days), and embedded in OCT™ (Tissue-Tek^®^, Sakura, Japan) on dry ice. Frozen sections of 10 or 20 µm thickness were cut using a cryostat (Leica CM1850; Leica Microsystems Ltd) and immunostained.

**Histochemistry**

Haematoxylin and eosin staining was performed on sections of methanol–acetic acid-fixed, paraffin-embedded brains. Sections were dewaxed in Histoclear™ (2 × 5 min) (National Diagnostics, Yorkshire, UK) and rehydrated to water through a decreasing ethanol series (2 × 3 min each in 100%; 3 min: 95%, 75%, 50%, 30%, water). Sections were stained for 5 min with Mayer’s Haematoxylin (Thermo Fisher Scientific, Loughborough, UK) and then rinsed in water followed by 0.0005% ammonia water (30 s) and water. After staining, sections were dehydrated through an increasing ethanol series (2 min: 30%, 50%, 70%) and counterstained in 1% Eosin-Y (Thermo Fisher Scientific), then in 70% ethanol for 2 min. The sections were dehydrated by passing through an ethanol series (3 s: 70%, 90%; 2 × 2 min: 100%), cleared in Histoclear™ (2 × 5 min; National Diagnostics), and mounted in DePeX™ mounting medium (Gurr; VWR International, Leuven, Belgium).

**Immunohistochemistry**

Sections were blocked in blocking buffer (PBS with 0.1% gelatin, 0.5% BSA, and 0.1% Tween-20) for 1–2 h. Sections were incubated with primary antibodies: mouse anti-calbindin (1:4000, 300; Swant, Marly, Switzerland); mouse anti-neurofilament (1:5, 2H3; DSHB, Iowa City, IA, USA); rabbit anti-pericentrin (1:2000, 448; Abcam, Cambridge, UK); rabbit anti-adenylyl cyclase III (1:1000, C-20; Santa Cruz, Dallas, TX, USA); rabbit anti-cleaved caspase-3 (1:2000, ab13847; Abcam); rabbit anti-Pax6 (1:500, PRB-278P; Covance, Dedham, MA, USA); mouse anti-BrdU (1:50, G3G4; DSHB); rabbit anti-phosphohistone 3 (1:1600, 3377; Cell Signaling Technology, Danvers, MA, USA); guinea pig anti-VGlut (1:6000, Ab5905; Millipore, USA); rabbit anti-GAD-65/67 (1:500, AB1511; Millipore, USA); and mouse anti-PCNA (1:4000, 2586; Cell Signaling Technology). Secondary antibodies used for indirect immunofluorescence were rabbit anti-mouse Alexa 546, goat anti-guinea pig Alexa 488, and goat anti-rabbit Alexa 488, all at 1:1000 dilution (Invitrogen, Waltham, MA, USA).

Antibodies were diluted in blocking buffer. Sections were incubated in the appropriate primary antibody overnight at 4°C. Sections were washed (3 × 10 min) in PBS with 0.1% Tween (PBST). Following the wash, the appropriate secondary antibody diluted in blocking buffer containing 4',6-diamidino-2-phenylindole (DAPI; 0.1 μg/ml; Sigma, St Louis, MO, USA) was applied to the sections and incubated for 2 h at room temperature. Sections were washed in PBST (3 × 10 min), followed by PBS (1 × 10 min), and mounted in Mowiol (Polysciences Inc, Hirschberg an der Bergstrasse, Germany). Images were acquired using a Leica DM55 microscope (Leica Microsystems Ltd) equipped with a DFC-6000 camera (Leica Microsystems Ltd) and processed using LAS software (Leica Microsystems Ltd).

**Proliferation analysis**

For quantification of proliferation in the EGL, a region of interest (ROI) spanning 200 μm length was selected from each cardinal lobe and the total numbers of DAPI^+^, BrdU^+^, and PH3^+^ cells were counted; because mice at P10/P15 exhibited fewer PH3^+^ cells, a larger ROI was used which encompassed the entire crown. ROIs were selected from three sections each from the brains of three mice. Statistical significance was assessed using Student’s *t*-test because data were normally distributed with equal variance. For quantification of proliferation and cell density in the IGL, a square ROI of 250 μm^2^ was selected and the total numbers of DAPI^+^, BrdU^+^, and PH3^+^ cells were counted. Data were normally distributed with equal variance and statistical significance was assessed using Student’s *t*-test.

**Quantification of layer thickness and apoptotic cells**

The thickness of the EGL was quantified from five equally spaced width measurements per cardinal lobe. Measurements were taken from three sections each from three mice. Data were not normally distributed and could not be improved by transformation, so the non-parametric Mann–Whitney test was used to assess significance.

Quantification of apoptotic cells in the EGL and IGL was carried out by counting the number of cleaved caspase-3-positive cells within each cardinal lobe of the mutant and wild-type mice (*n* = 3). The total area of the cardinal lobe was first measured and the number of cleaved caspase-3-positive cells per 10 000 μm^2^ was calculated. Data from the IGL were normally distributed with equal variance; thus, Student’s *t*-test was used to show significance. Data from the EGL were not normally distributed and could not be improved by transformation, so the non-parametric Mann–Whitney test was used to test statistical significance.

**Purkinje cell analysis**

Multiple photomicrographs were obtained of Purkinje cells stained for calbindin and stitched together using Fiji imaging software [26]. The total number of Purkinje cells was counted in three mid-sagittal sections from *Talpid3* mutant and wild-type mice (*n* = 3). The total cerebellar area was measured from each section and Purkinje cell density was calculated. Log_10_ transformation was able to equalise the variance between the samples. The transformed data were analysed using Student’s *t*-test.

Primary and total dendrite length were measured from 26–30 Purkinje cells taken from two to four sections each from mutant and wild-type mice (*n* = 3). Data were normally distributed with equal variance and Student’s *t*-test was used to test for statistical significance.

**Quantification of proliferating cells**

The numbers of proliferating cells were quantified from images of brain sections stained immunohistochemically for either PCNA and PH3 or BrdU and PH3 using Fiji imaging software [26]. For each anatomical region, three independent fields of comparable axial level and area were selected per mouse (*n* = 3). In each field, the numbers of PH3^+^ and PCNA/BrdU^+^ nuclei were counted. Autofluorescent cells were identified by a bright yellow colour in overlaid images and were not counted. The numbers of PH3^+^ and PCNA/BrdU^+^ nuclei were expressed as a percentage of the total number of nuclei stained with DAPI in each region [(No of PH3^+^ or PCNA/BrdU^+^ nuclei/total number nuclei) × 100]. Percentages from each field were used to calculate average and standard deviation values for each anatomical region. Cell density was calculated by dividing the total number of cells by the total area of tissue used for cell counting and was expressed as number of cells per 10 000 µm^2^. Cell densities from each field were used to calculate average and standard deviation values for each anatomical region.

**Image analyses for estimation of nuclear shape and orientation**

Analyses of nuclear shape and orientation from stained sections were performed using Fiji imaging software [26] from an ROI spanning a 50 μm length of the EGL. All images were rotated to adjust the EGL to a horizontal orientation and nuclei were outlined. Orientation was calculated by drawing an ellipse of best fit and using the major angle ranging from 0 to 180°. All angles greater than 90° were mirrored using the following equation: $\sqrt{[ {x-180]}^{2}}$. Data from individual nuclei were pooled between fields and mice (*n* = 3). Data were considered normal using the Anderson–Darling test for normality. Student’s *t*-test was used to assess statistical significance.

**RNA isolation, cDNA synthesis, and qPCR analysis of signalling pathway components**

P15 cerebella were dissected, cut into small pieces, and frozen in Trizol reagent (Invitrogen) using liquid nitrogen. Tissues were thawed; homogenised by passing through 20-, 25-, and 30-gauge needles; and total RNA was extracted according to the manufacturer’s instructions. RNA was treated with a DNA-free Kit (Thermo Fisher Scientific) and its quality and quantity were assessed using an Experion RNA StdSens Analysis kit (Bio-Rad Laboratories Inc, Watford, UK); all RNAs had an RQI value greater than 8. RNA was annealed to oligo-dT primers and cDNA synthesis was completed using RevertAid H Minus Reverse Transcriptase (Thermo Fisher Scientific). Duplicate reactions omitting reverse transcriptase enzyme were completed as wild type. Reactions were performed at 42°C for 60 min and the reverse transcriptase was inactivated at 70°C for 5 min. Reactions were diluted in DEPC-H_2_O to a concentration of 10 ng/μl.

A mastermix comprising iQ SYBR Green supermix (Bio-Rad Laboratories Inc), water, and cDNA was made for quantitative PCR. Appropriate primers were added and divided into three replicates for each gene on PCR plates (Thermo Fisher Scientific). Final reactions were in 20 µl volumes with 0.1 µm primers and 10 ng of cDNA. Reactions were carried out in a BioRad iQ5 cycler. Dynamic well factors were collected for 2.5 min, followed by 40 cycles at 60°C and 95°C for 20 s each, and then by a melt curve. RT-minus wild types were run only for *Gapdh* primers as all other primers were intron-spanning. Expression levels were quantified relative to *Gapdh* and comparisons were made between three or four control and mutant mice (*n* = 3 or *n* = 4). Student’s *t*-test was used to compare mean expression values between control and mutant mice. Primer sequences and product size are listed in the supplementary material, Table S1.

**Western blotting**

Cerebella were dissected from the brain, washed in cold PBS, and minced before addition of lysis buffer (2% SDS in 50 mm Tris, pH 6.8). Samples were homogenised and passed through 21 G needles. The homogenates were centrifuged at 16 000 × *g* for 5 min and cleared supernatants were aliquoted and stored frozen at −80°C. Protein concentration of thawed samples was estimated using a BCA Protein Assay Kit (Pierce, Thermo Fisher Scientific). For PAGE, loading buffer was added to 20 μg of lysed protein to a final concentration of 2% SDS, 10% glycerol, 100 mm DTT, and 0.01% bromophenol blue in 60 mm Tris, pH 6.8. Samples were boiled for 5 min and then electrophoresed through 7% Tris–glycine gels (National Diagnostics) under denaturing conditions. Proteins were transferred to PVDF membranes (Millipore) at 50 V for 2 h in transfer buffer (25 mm Tris; 192 mm glycine, pH 8.3; 20% methanol; 0.05% SDS). Membranes were blocked with blocking buffer (PBS, 5% Marvel milk powder, and 0.1% Tween-20) for 1 h. Blots were incubated overnight at 4°C with the following primary antibodies diluted in blocking buffer: anti-Gli2 (Ab26056, 1:1000; Abcam) and anti-Gli3 (H280, 1:500; Santa Cruz). Membranes were washed in PBS containing 0.1% Tween-20 (4 × 15 min) and incubated with HRP-conjugated anti-mouse secondary antibody (1:10 000; Abcam) for 1 h. Membranes were washed twice in PBS–Tween and twice in PBS before incubating in ECL reagent (Pierce, Thermo Fisher Scientific). Chemiluminescence was detected using a Fusion Imaging system (Vilber Lourmat, Collégien, France). Protein sizes were estimated using relative mobility values calculated from electrophoresed samples of the Page Ruler Unstained Broad Range Protein Ladder (Thermo Fisher Scientific).
