# Supplementary material for: Mice with a conditional deletion of Talpid3 (KIAA0586) – a model for Joubert syndrome
Source: J Pathol. 2019 May 16;248(4):396–408. doi: 10.1002/path.5271 (PMC6767539; doi:10.1002/path.5271)
Supplement: Supplementary file 2 — Supplementary video legend [file PATH-248-396-s002.docx]

**Mice with a conditional deletion of *Talpid3* (*KIAA0586*) – a model for Joubert syndrome**

Bashford AL *J Pathol* DOI: 10.1002/path.5271

**Video S1.** *Ta3* mutant mice exhibit severe ataxia but maintain grooming behaviour.

(A) ‘Ataxia’ – P15 control and *Ta3* mutant littermates moving around the cage. The *Ta3* mutant mouse attempts purposeful movement but is unable to coordinate limbs or maintain balance (B) ‘Grooming’ – at P15, the *Ta3* mutant mouse displays limited motor control but maintains its ability to feed and groom.
