# Supplementary material for: Mice with a conditional deletion of Talpid3 (KIAA0586) – a model for Joubert syndrome
Source: J Pathol. 2019 May 16;248(4):396–408. doi: 10.1002/path.5271 (PMC6767539; doi:10.1002/path.5271)
Supplement: Supplementary file 3 — Figure S1. Growth and foliation of cerebella in Ta3 mutant and wild‐type mice Figure S2. Cell density and features of the EGL and IGL of the Ta3 mutant and wild‐type cerebella Figure S3. Primary cilia in Ta3 mutant and wild‐type cerebella Figure S4. Cell proliferation in E18.5 Ta3 mutant and wild‐type cerebella Figure S5. Apoptosis in the cerebella of Ta3 mutant and wild‐type mice Figure S6. Orientation of nuclei/cells in Ta3 mutant EGL Figure S7. Bergmann glia are misplaced in the Ta3 mutant cerebellum Figure S8. Morphology of the PCL, PCs, and dendritic arborisation in Talpid3 mutant and wild‐type cerebella [file PATH-248-396-s003.docx]

**Mice with a conditional deletion of *Talpid3* (*KIAA0586*) – a model for Joubert syndrome**

Bashford AL *J Pathol* DOI: 10.1002/path.5271

**Supplementary figures**


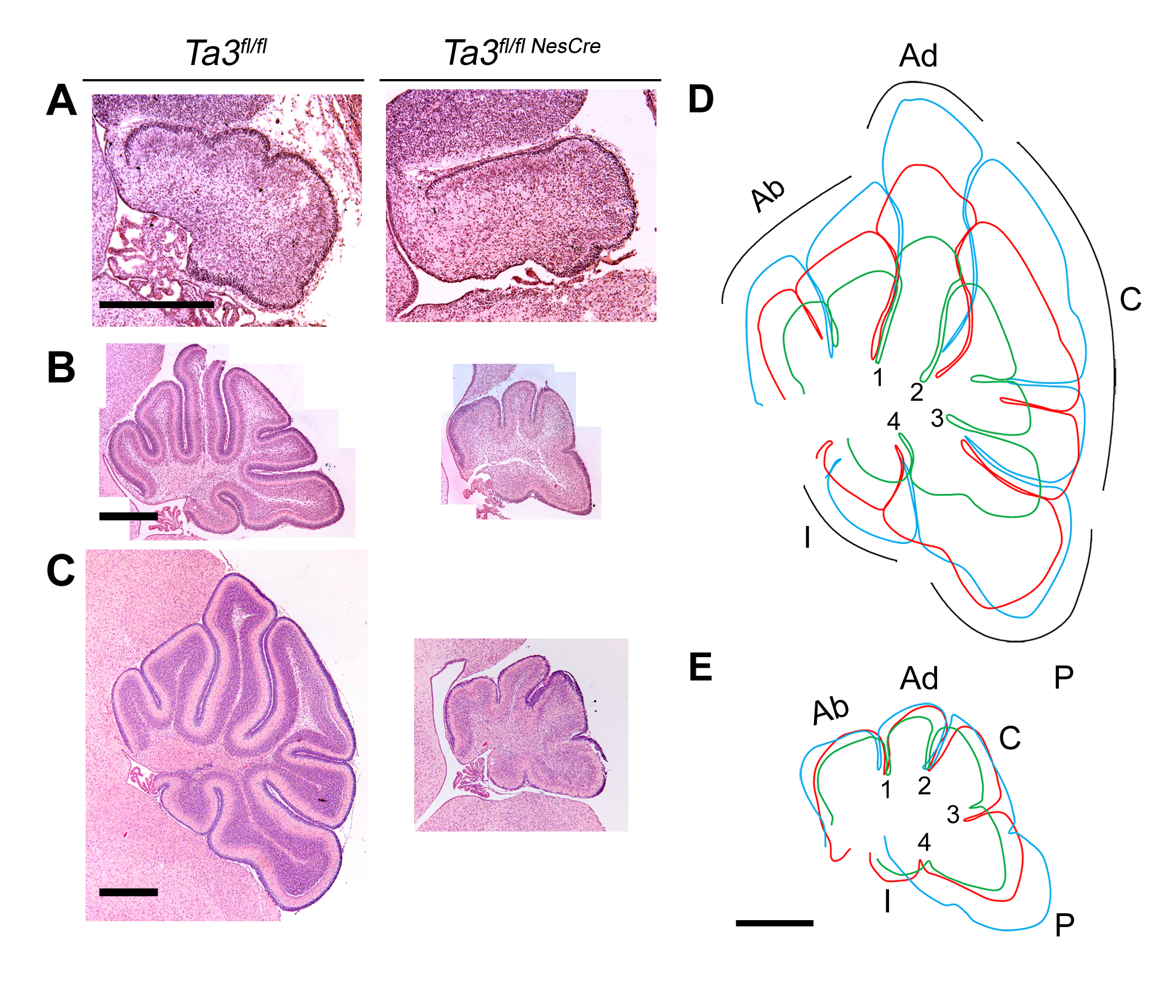


**Figure S1. Growth and foliation of cerebella in *Ta3* mutant and wild-type mice.**

*Ta3* mutant cerebella at E18 (A), P5 (B), and P10 (C) have reduced foliation and are smaller in size than wild type. (D) Wild-type cerebella show consistent growth between P5 (green), P10 (red), and P15 (blue). (E) *Talpid3* mutant cerebella show little growth and only exhibit the four principal fissures and five cardinal lobes. *n* = 3. Ab, anterobasal; Ad, anterodorsal; C, central; P, posterior; I, inferior. Scale bars: 500 μm (A–E).


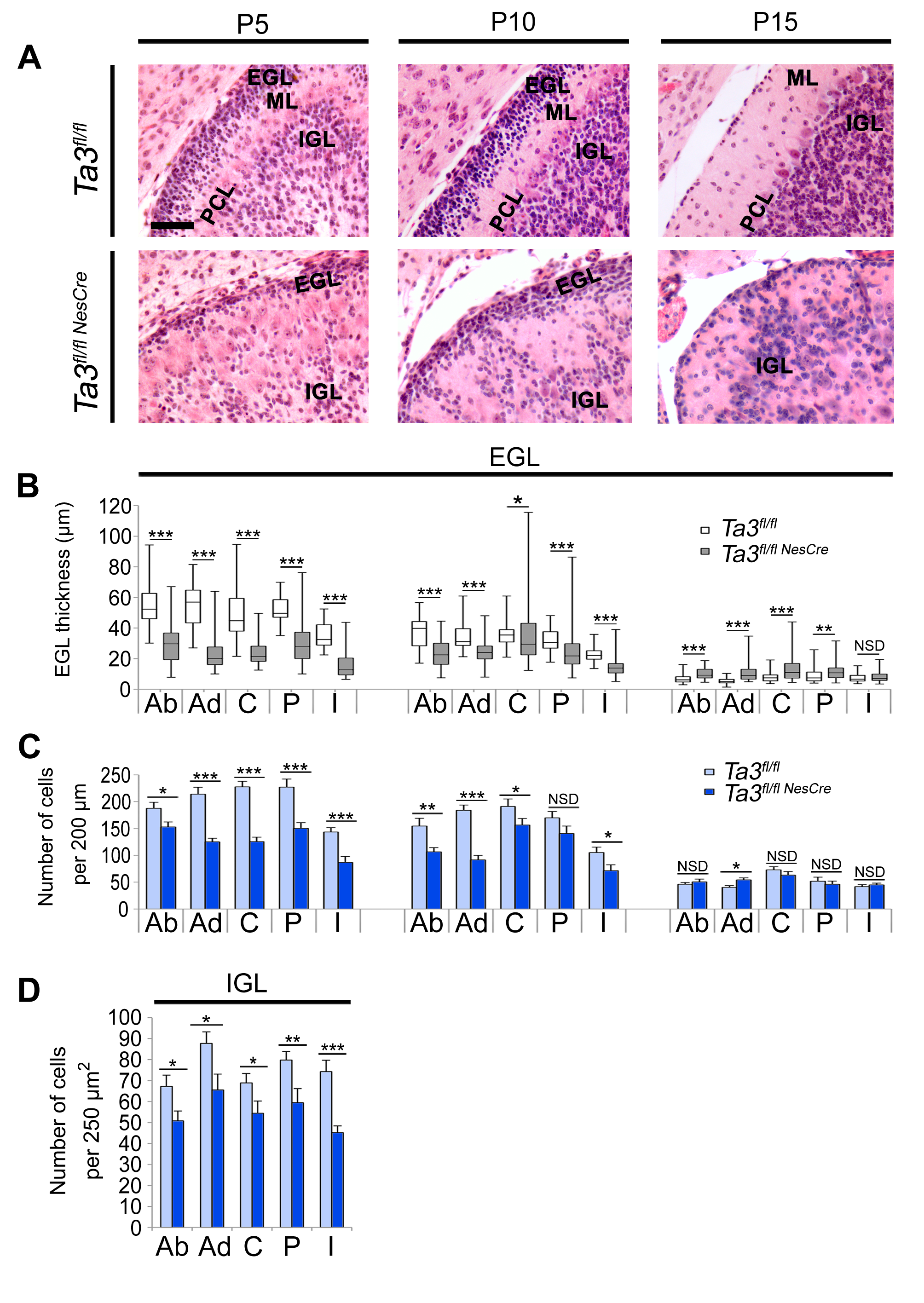


**Figure S2. Cell density and features of the EGL and IGL of the *Ta3* mutant and wild-type cerebella.**

(A) H&E-stained sections of control and *Ta3* mutant cerebella at P5, P10, and P15. (B) Quantification of thickness of the EGL in control and *Ta3* mutant mice at P5, P10 and P15. (C, D) Quantification of cell density in the EGL and IGL of control and *Ta3* mutant mice. Ab, anterobasal; Ad, anterodorsal; C, central; EGL, external granule layer; I, inferior; IGL, internal granule layer; P, posterior. Error bars: (B–D), SEM (*n* = 3), NSD = no significant difference; ****p* ≤ 0.001, ***p* ≤ 0.01, **p* < 0.05 (one-tailed Student’s *t*-test). (B) Box plot. ****p* < 0.001, ***p* < 0.01, **p* < 0.05 (one-tailed Mann–Whitney test). Scale bars: 50 µm.


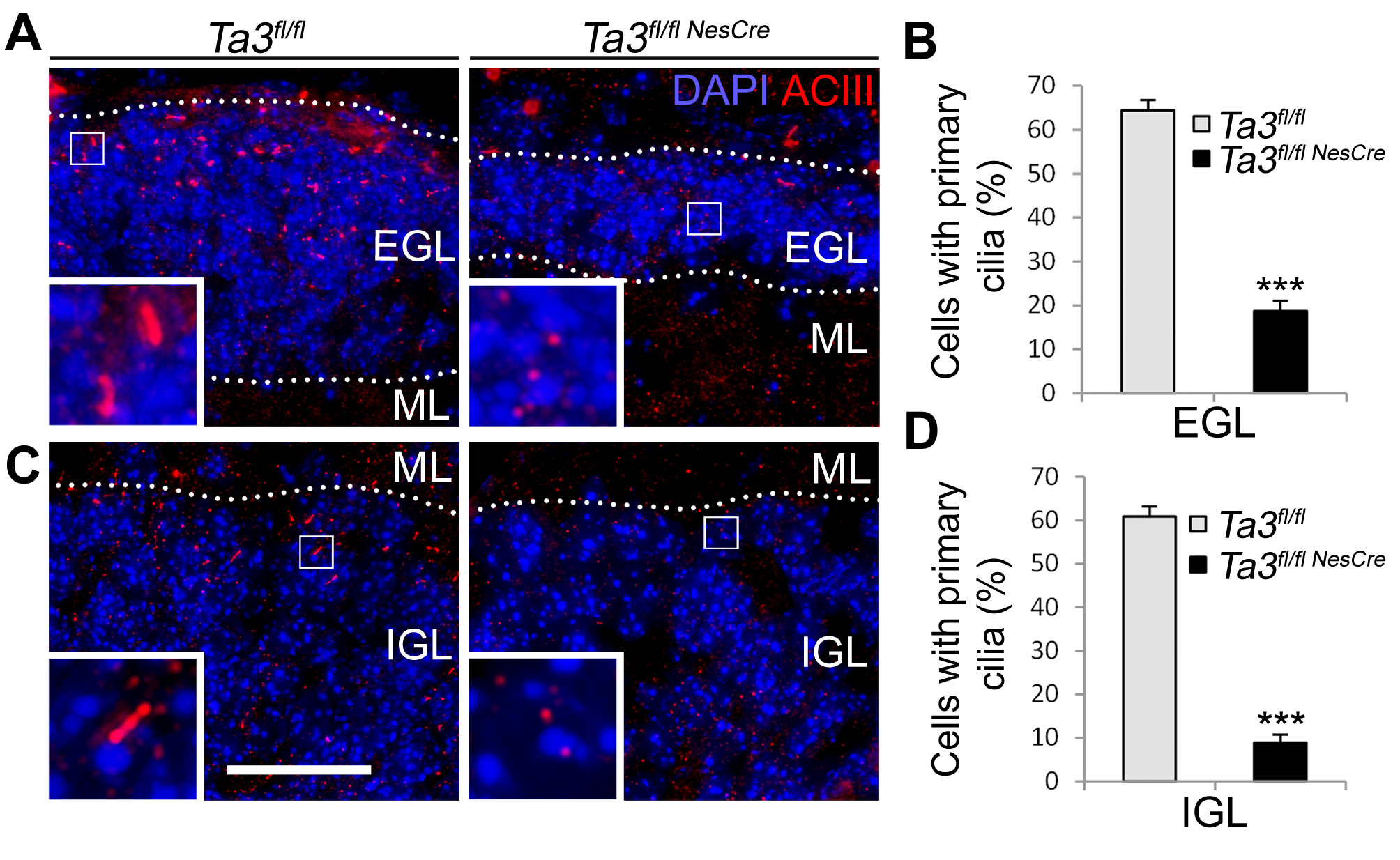


**Figure S3. Primary cilia in *Ta3* mutant and wild-type cerebella.**

Primary cilia immunostained for adenylyl cyclase III in P5 wild-type and *Ta3* mutant cerebella. (A) EGL and (C) IGL. Inset: greater magnification of the boxed area showing a well-formed cilia in wild type. (B, D) Quantification of cilia numbers in the EGL and in the outer 50 μm of the IGL. Dotted lines indicate EGL and IGL boundaries. EGL, external granule layer; ML, molecular layer; IGL, internal granule layer. Error bars (B, D), SEM (*n* = 3). ****p* < 0.001 (one-tailed Student’s *t*-test). Scale bars: 25 µm (A, C).


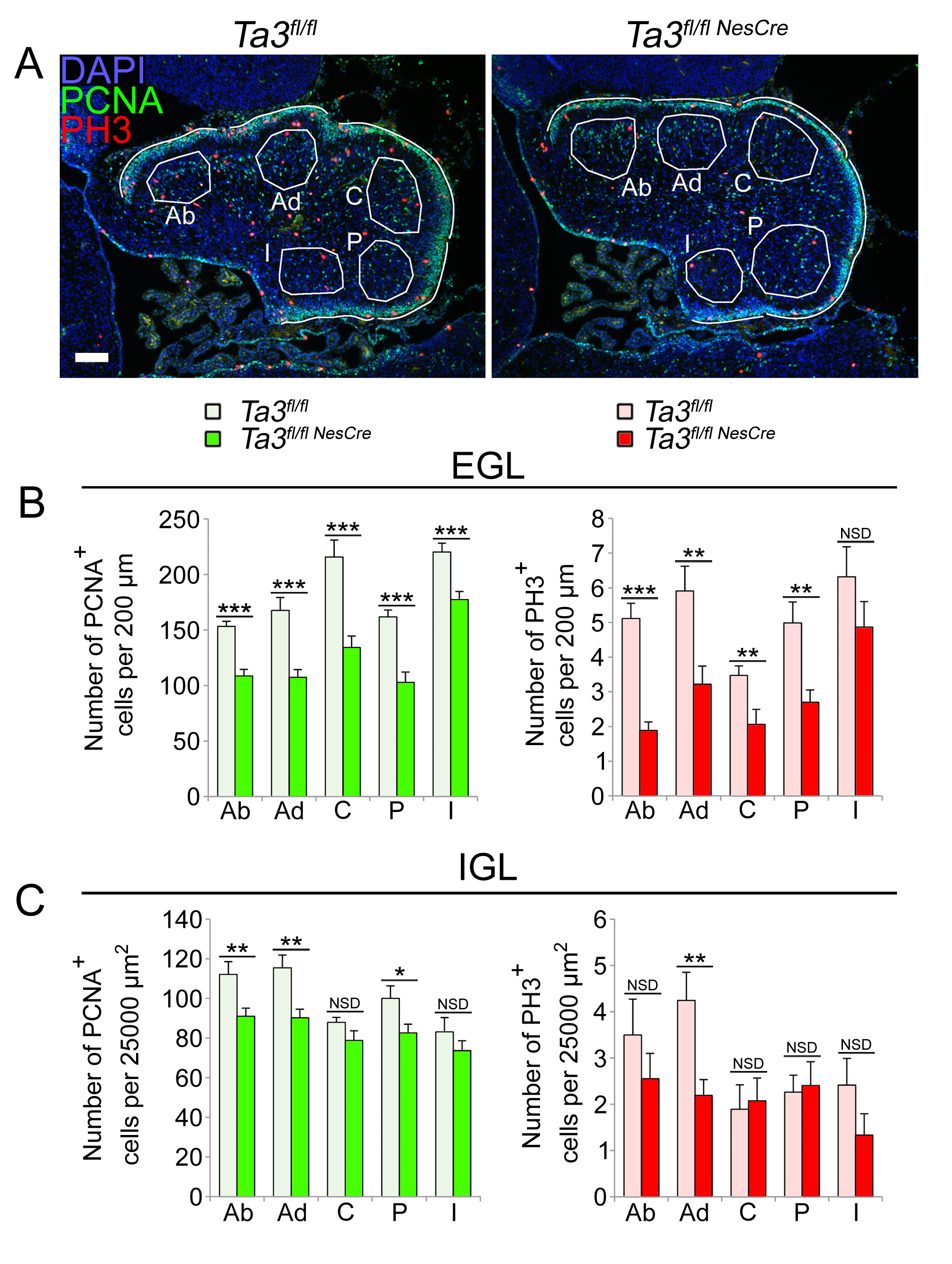


**Figure S4. Cell proliferation in E18.5 *Ta3* mutant and wild-type cerebella.**

(A) E18 control and *Ta3* mutant cerebella were stained for PCNA and PH3 to identify proliferating cells. Quantification of proliferating cell numbers in (B) the EGL and (C) the IGL of control and *Ta3* mutant cerebella. Ab, anterobasal; Ad, anterodorsal; C, central; EGL, external granule layer; I, inferior; IGL, internal granule layer; P, posterior; PCNA, proliferating cell nuclear antigen; PH3, phosphohistone 3. Error bars in B and C – SEM (*n* = 3), NSD = no significant difference; ****p* ≤ 0.001, ***p* ≤ 0.01, **p* < 0.05 (one-tailed Student’s *t*-test). Scale bar: 100 µm (A).


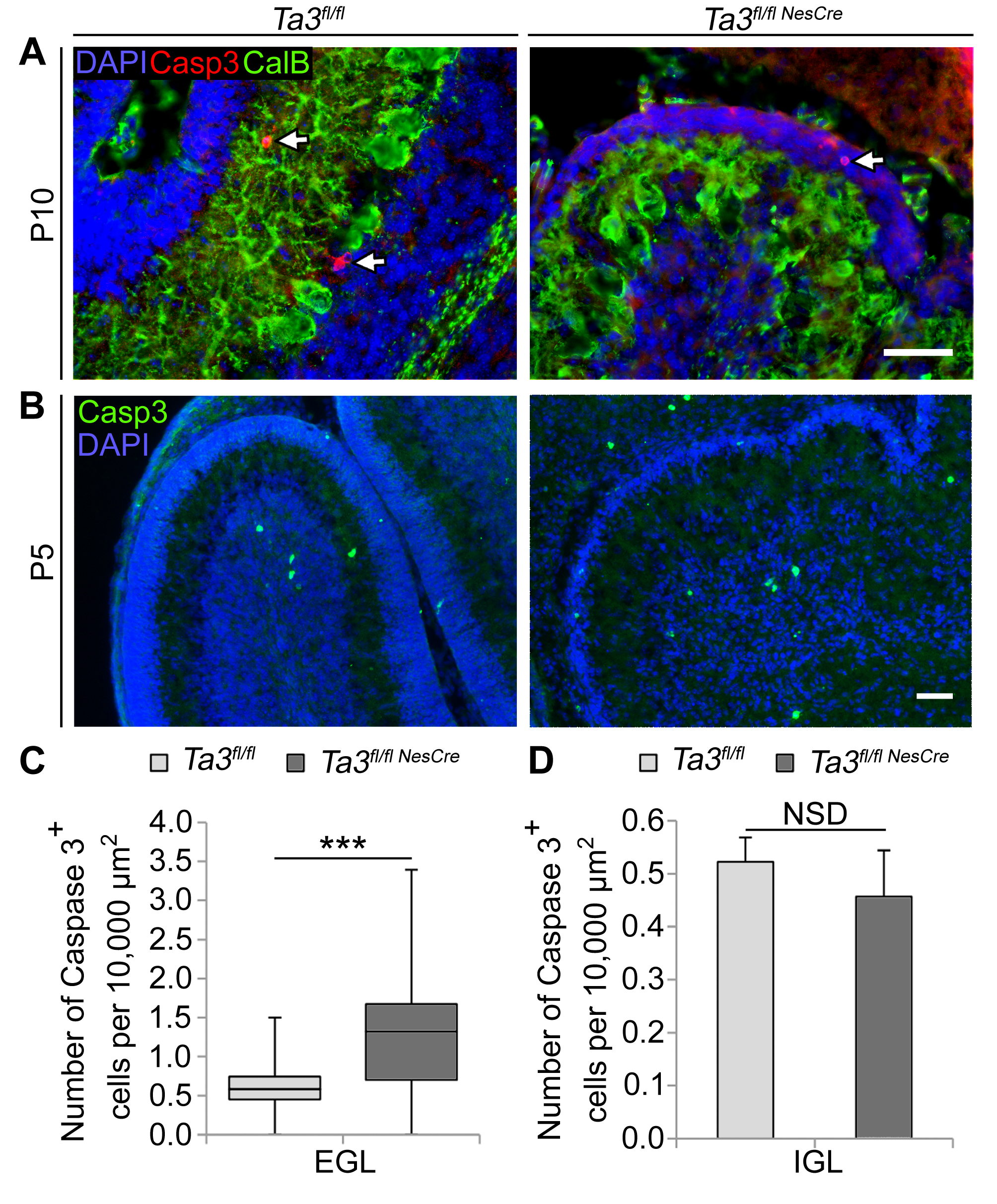


**Figure S5. Apoptosis in the cerebella of *Ta3* mutant and wild-type mice.**

(A, B) Control and *Ta3* mutant P10 and P5 cerebella stained for activated (cleaved) caspase-3. Number of caspase-3-positive cells in (C) EGL and (D) IGL. Casp3, cleaved caspase-3; CalB, calbindin D-28k; EGL, external granule layer; IGL, internal granule layer. Error bars (C) Box plot (*n* = 3), ****p* = 0.001 (one-tailed Mann–Whitney test); (D) SEM (*n* = 3), NSD = no significant difference (two-tailed Student’s *t*-test). Scale bar: 50 μm (A, B).


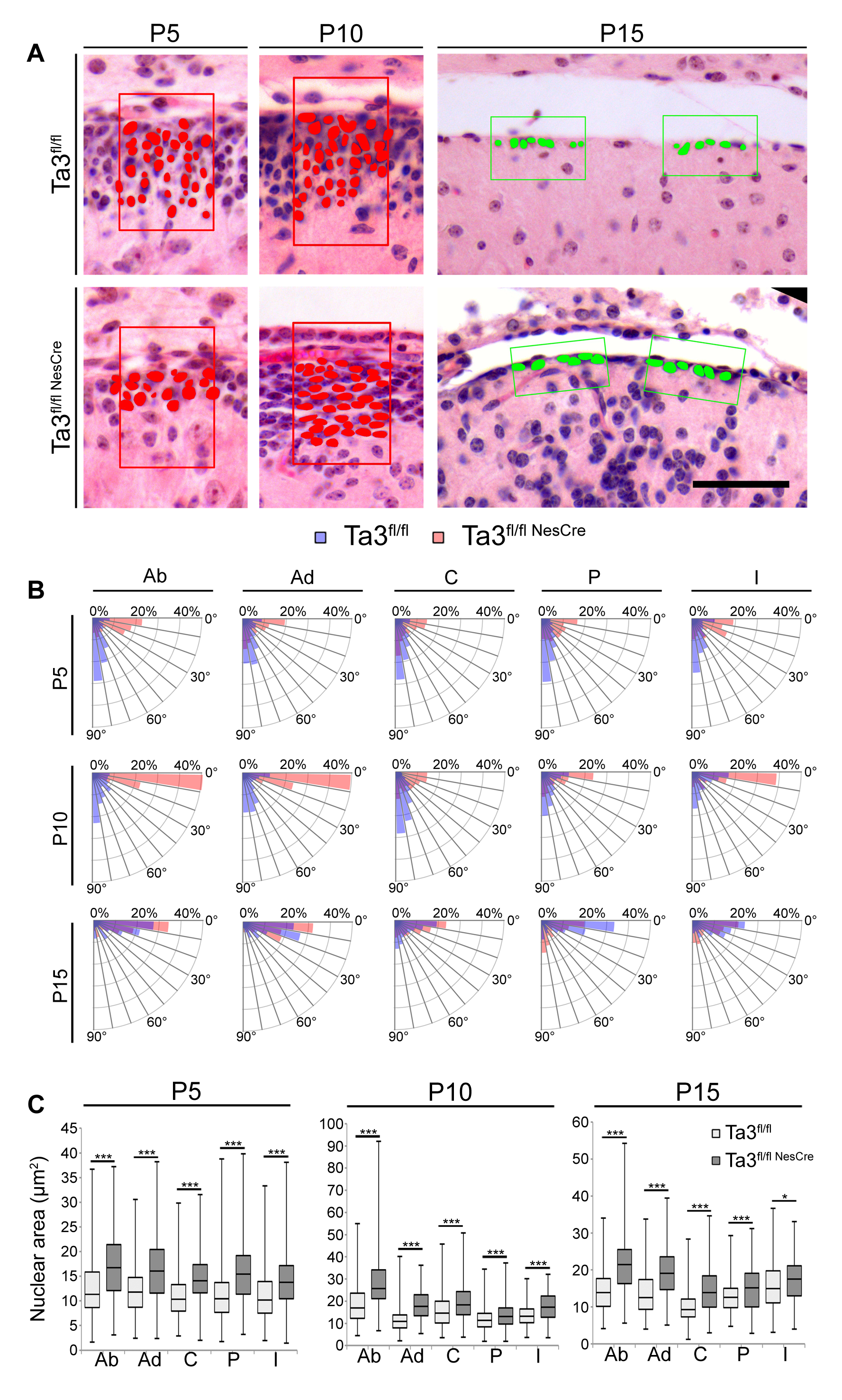


**Figure S6. Orientation of nuclei/cells in *Ta3* mutant EGL.**

(A) Representative images of P5, P10, and P15 cerebella showing the EGL of the anterobasal fold. Boxed nuclei filled in red/green illustrate sample used for analysis. (B) Radial plot showing frequency of nuclear orientations. Blue bars: WT; red bars: *Ta3* mutant. Angle of bar indicates orientation within 10° bins (0° = parallel with EGL; 90° = perpendicular to EGL). Length of bar indicates percentage frequency. (C) Box plots showing median nuclear area of P5, P10, and P15 EGL. (C) Box plot, ****p* < 0.001; **p* < 0.05 (one-tailed Mann–Whitney test). Scale bars: 50 µm.


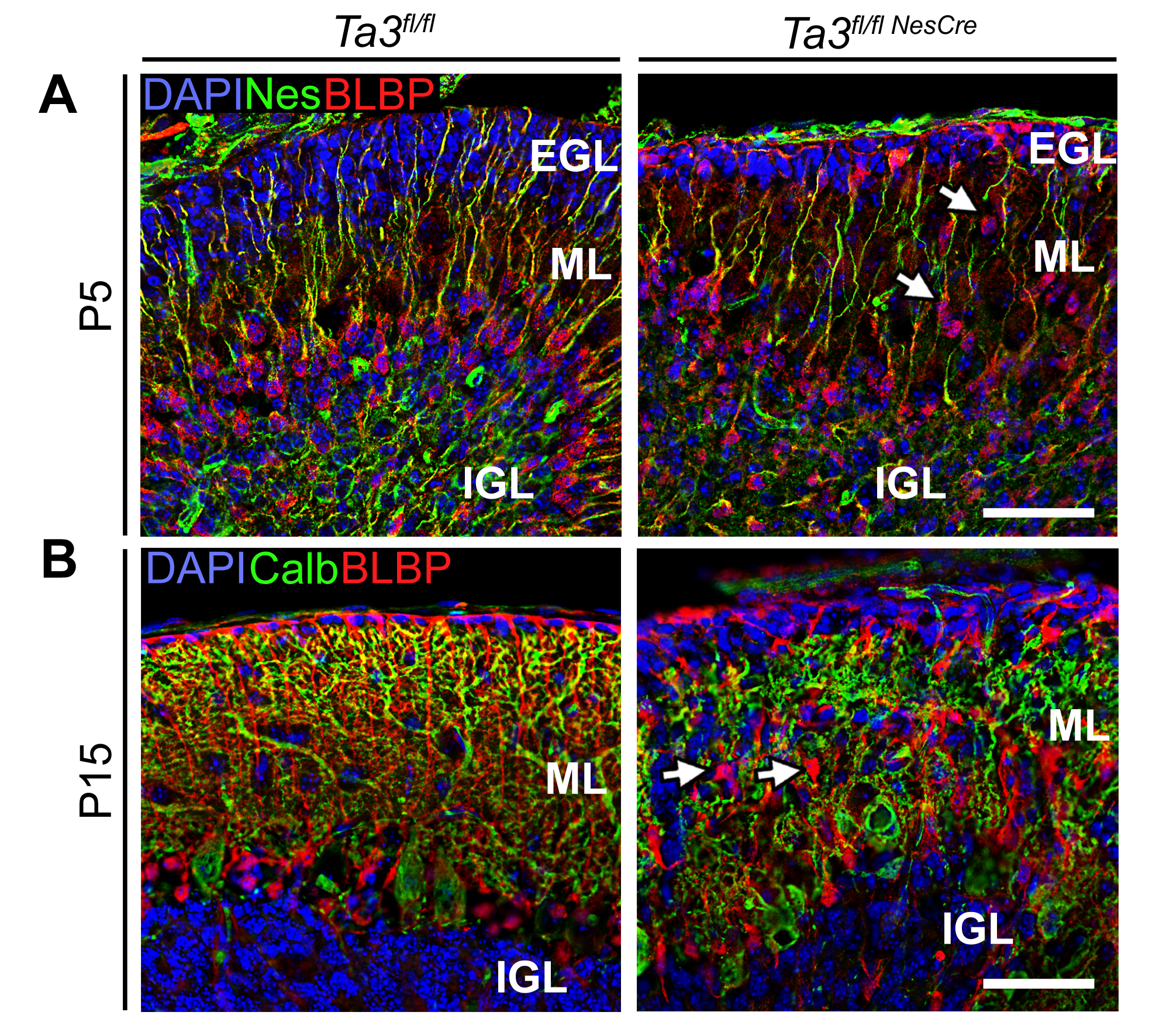


**Figure S7. Bergmann glia are misplaced in the *Ta3* mutant cerebellum.**

(A) P5 cerebella showing Bergmann glia immunostained for Nestin (green) and BLBP (red). Mutants exhibit fewer radial fibres and misplaced Bergmann glia in the ML (white arrow). (B) P15 cerebella immunostained for calbindin D-28k (green) and BLBP (red). *Ta3* mutant cerebella have mislocalised Bergmann glia (white arrow) with loss of end-feet in the EGL. Scale bars: 50 μm.


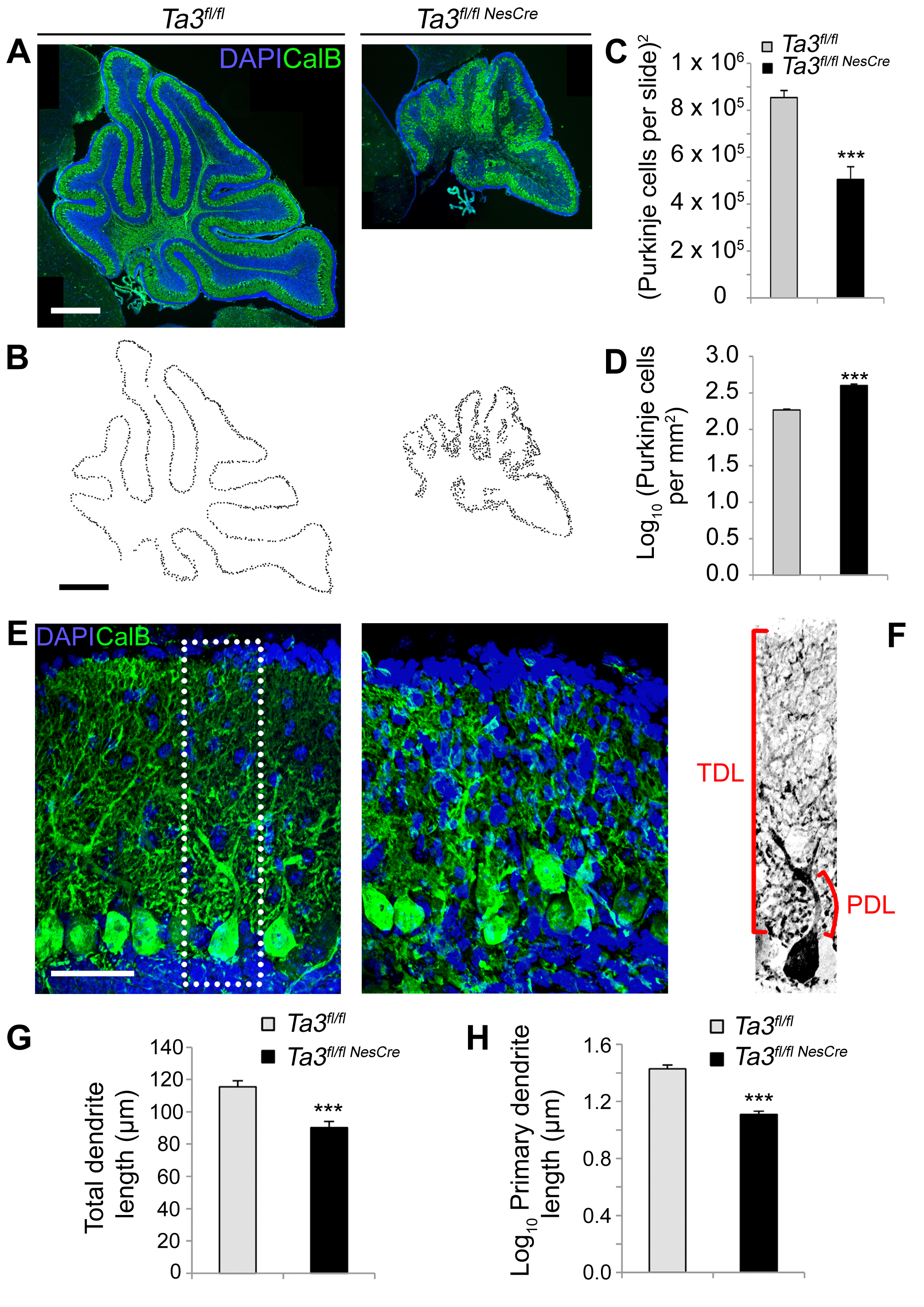


**Figure S8. Morphology of the PCL, PCs, and dendritic arborisation in *Talpid3* mutant and wild-type cerebella.**

(A) P10 wild-type and *Talpid3* mutant cerebella immunostained for calbindin. (B) Trace of Purkinje cells reveals the extent of disorganisation of the PCL and clusters of PCs at the base of the folds. (C) Quantification of Purkinje neurons per section in wild-type and *Talpid3* mutants. Data transformed (squared) to equalise sample variance. (D) Density of Purkinje neurons in wild-type and *Talpid3* mutant cerebella. Data transformed (log_10_) to equalise sample variance. (E) Dendritic arborisation of wild-type and *Talpid3* mutant Purkinje neurons in P15 cerebella. (F) Total dendrite length and primary dendrite length from the boxed region in E shown as a trace image. (G) Quantification of wild-type and *Talpid3* mutant Purkinje neurons total dendrite length and (H) primary dendrite length. Data transformed (log_10_) to equalise sample variance. PC, Purkinje cells; PCL, Purkinje cell layer; CalB, calbindin; PDL, primary dendrite length; TDL, total dendrite length. Error bars (C, D) SEM (*n* = 3), ****p* < 0.001 (one-tailed Student’s *t*-test). (G) SEM (data from 30 Purkinje cells), ****p* < 0.001 (one-tailed Student’s *t*-test). (H) SEM (data from 26 Purkinje cells), ****p* < 0.001 (one-tailed Student’s *t*-test). Scale bar: 500 µm (A, B); 50 µm (E).
