# Supplementary material for: Mice with a conditional deletion of Talpid3 (KIAA0586) – a model for Joubert syndrome
Source: J Pathol. 2019 May 16;248(4):396–408. doi: 10.1002/path.5271 (PMC6767539; doi:10.1002/path.5271)
Supplement: Supplementary file 4 — Table S1. Primer sequences for qPCR [file PATH-248-396-s004.docx]

**Mice with a conditional deletion of *Talpid3* (*KIAA0586*) – a model for Joubert syndrome**

Bashford AL *J Pathol* DOI: 10.1002/path.5271

**Table S1.** Primer sequences for qPCR

| **Gene symbol** | **Primer sequence** | **Amplicon size (bp)** |
| --- | --- | --- |
| *Gapdh* | FP – 5' CCTTCCGTGTTCCTACCCCCAATG 3'  RP – 5' GGAGACAACCTGGTCCTCAGTGTA 3' | 155 |
| *Gli1* | FP – 5' TTCGTGTGCCATTGGGGAGG 3'  RP – 5' CTTGGGCTCCACTGTGGAGA 3' | 440 |
| *Gli2* | FP – 5' TTCGTGTGCCGCTGGCAGGC 3'  RP – 5' TTGAGCAGTGGAGCACGGAC 3' | 425 |
| *Gli3* | FP – 5'TTCGTGTGCCGCTGGCTTGA 3'  RP – 5' TGAATGGCTGCCGGAATCTC 3' | 444 |
| *Ptch1* | FP – 5' GGTCACACGAACAATGGGTCT 3'  RP – 5' CACATTCCACGTCCTGTAGC 3' | 682 |
| *Smo* | FP – 5' TGGGATCCAGTGCCAGAACCCGCT 3'  RP – 5' ACGGTACCGATAGTTCTTGTAGCC 3' | 562 |
| *Axin2* | FP – 5' GCGACGCACTGACCGACGAT 3'  RP – 5' GCAGCAGGTTCCACAGGCGT 3' | 196 |
| *Dll1* | FP – 5' GGTTGCTCTGTGTTCTGCCG 3'  RP – 5' GTTGGTCATCACACCCTGGC 3' | 142 |
| *Dll3* | FP – 5' CTGGACCTTGTGATGGGAACC 3'  RP – 5' CTCACCTCACATCGAAGCCC 3' | 112 |
| *Hes1* | FP – 5' GAGCACAGAAAGTCATCAAAGCC 3'  RP – 5' TCTCTAGCTTGGAATGCCGG 3' | 133 |
| *Hes5* | FP – 5' GAGATGCTCAGTCCCAAGGAG 3'  RP – 5' GCGAAGGCTTTGCTGTGTTT 3' | 203 |
| *Notch1* | FP – 5' CGTGGATTCATCTGTAGGTGC 3'  RP – 5' CATAGGCAGGTGGGACTACG 3' | 134 |
| *Notch2* | FP – 5' GCTGTCAATAATGTGGAGGCG 3'  RP – 5' TTGGCCGCTTCATAACTTCC 3' | 125 |
| *Wnt3a* | FP – 5' CAAGCACAACAATGAAGCAGGC 3'  RP – 5' TCGGGACTCACGGTGTTTCTC 3' | 199 |
| *Wnt7a* | FP – 5' CGACTGTGGCTGCGACAAG 3'  RP – 5' CTTCATGTTCTCCTCCAGGATCTTC 3' | 200 |

FP: forward primer; RP: reverse primer.
